# The Hypolipidemic and Pleiotropic Effects of Rosuvastatin Are Not Enhanced by Its Association with Zinc and Selenium Supplementation in Coronary Artery Disease Patients: A Double Blind Randomized Controlled Study

**DOI:** 10.1371/journal.pone.0119830

**Published:** 2015-03-18

**Authors:** Karine Cavalcanti Maurício Sena-Evangelista, Lucia Fatima Campos Pedrosa, Maria Sanali Moura Oliveira Paiva, Paula Cristina Silveira Dias, Diana Quitéria Cabral Ferreira, Sílvia Maria Franciscato Cozzolino, Tanize Espírito Santo Faulin, Dulcinéia Saes Parra Abdalla

**Affiliations:** 1 Department of Nutrition, Federal University of Rio Grande do Norte, Natal, RN, Brazil; 2 Onofre Lopes University Hospital, Federal University of Rio Grande do Norte, Natal, RN, Brazil; 3 Post-Graduate Program in Pharmaceutical Sciences, Federal University of Rio Grande do Norte, Natal, RN, Brazil; 4 Department of Food and Experimental Nutrition, Faculty of Pharmaceutical Sciences, University of São Paulo, São Paulo, SP, Brazil; 5 Department of Clinical and Toxicological Analyses, Faculty of Pharmaceutical Sciences, University of São Paulo, São Paulo, SP, Brazil; University of Insubria, ITALY

## Abstract

**Objective:**

Statins treatment may modify the levels of zinc and selenium, minerals that can improve vascular function and reduce oxidative damage and inflammation in atherosclerotic patients. This study aimed to evaluate the effects of rosuvastatin, alone or associated with zinc and selenium supplementation, on lipid profile, antioxidant enzymes and mineral status in coronary artery disease patients.

**Material and Methods:**

A double-blind randomized clinical trial was performed in which patients (n = 76) were treated with 10 mg rosuvastatin over 4 months associated or not with zinc (30 mg/d) and selenium (150 μg/d) supplementation. The following parameters were analyzed before and after the intervention: anthropometric measurements, lipid profile, high sensitivity C-reactive protein (hs-CRP), electronegative low density lipoprotein (LDL(-)) concentrations, activities of glutathione peroxidase (GPx), superoxide dismutase (SOD), zinc and selenium concentrations in blood plasma and erythocytes. Significance was determined using an α of 5% (two-tailed).

**Results:**

We found that rosuvastatin therapy was efficient in reducing total cholesterol, LDL-cholesterol, non-HDL cholesterol, triglycerides, and hs-CRP independently of mineral supplementation. Neither treatment was associated with significant changes in LDL(-). Similarly, the antioxidant enzymes GPx and SOD activity were unchanged by treatments. Neither treatment was associated with significant differences in concentrations of zinc or selenium in blood plasma and erythocytes of studied groups.

**Conclusion:**

Rosuvastatin treatment did not affect zinc and selenium levels in coronary artery disease patients. The zinc and selenium supplementation at doses used in this study did not change lipid profile or SOD and GPx activity in patients receiving rosuvastatin. Further studies should be focused on testing alternative doses and supplements in different populations to contribute for a consensus on the ideal choice of antioxidants to be used as possible complementary therapies in atherosclerotic patients.

**Trial Registration:**

ClinicalTrials.gov NCT01547377

## Introduction

Zinc and selenium are important minerals for maintaining endothelial integrity as they inhibit events related to oxidative stress and inflammation [1,2]. Zinc, which acts as an antioxidant and stabilizes cell membranes, has a protective effect on the vascular endothelium. Moreover, zinc plays a vital role on the activities of nuclear factor *Kappa* B (NF-κB), caspases, and nitric oxide synthase, besides to reduce infection rates [3]. Selenium also interacts with NF-κB and decreases the activity of this transcription factor during inflammatory response in endothelium, thereby helping to prevent atherosclerosis [4].

Concentrations of trace elements may be reduced in patients with cardiovascular diseases due to metabolism alterations and increased nutritional requirements. However, zinc and selenium bioavailability alterations have been attributed to the use of statins [5,6,7,8]. These drugs have antiatherosclerotic effects that positively correlate with the decrease of low density lipoprotein cholesterol (LDL-c). In addition, they can exert beneficial pleiotropic effects, including improvement of endothelial function, increased stability of atherosclerotic plaques, inhibition of the thrombogenic response, and decrease of inflammation and oxidative stress [9].

Reports on compromised mineral status and the use of statins, particularly rosuvastatin, are scarce. It was previously reported that the treatment of hypercholesterolemic patients with fluvastatin (80 mg/day for 8 weeks) decreased zinc plasma concentrations [5]. Likewise, decrease of serum zinc and copper concentrations has been observed in dyslipidemic patients treated with simvastatin or atorvastatin (10 mg/day for 4 months). Although a significant change of zinc was observed only in the simvastatin group, neither group experienced changes in serum selenium concentrations [10].

There are scarce data regarding the zinc and selenium status in patients with stable angina undergoing treatment with rosuvastatin. Mainly, no information is available regarding whether concomitant treatment with rosuvastatin and zinc/selenium supplements improves antioxidant capacity or alters the concentrations of these minerals in the organism. This is important, considering that supplements may provide benefits to specific subpopulations with regards to meeting their nutritional needs according to science-based standards for example, nutritional older adults who have inadequate dietary and those with medical conditions that limit food choices [11]. It is often recommend that patients take non-toxic, supplementary doses of zinc and selenium (either in combination or separately) in order to maintain adequate levels of these minerals [12].

Thus, the current study was designed to compare the effects of rosuvastatin treatment alone with a combination treatment of rosuvastatin and oral zinc and selenium supplementation, on lipid profile, antioxidant enzymes and mineral status in coronary artery disease patients. The results of this study can be used to direct supplementation strategies during clinical treatment of coronary artery disease patients.

## Materials and Methods

The protocol for this trial and supporting CONSORT checklist are available as supporting information; see S1 CONSORT Checklist and S1 Protocol.

### Ethics Statement

The study was approved by the Research Ethics Committee of the Onofre Lopes University Hospital and the Faculty of Pharmaceutical Sciences of the University of São Paulo (CAAE number 0009.0.294.018-06 – SISNEP), according to official documents in Brazil (National Health Council 466/2012). All participants provided written informed consent. This study was registered at http://www.clinicaltrials.gov as NCT01547377. We choose to register the study after getting all the results. The authors confirm that all ongoing and related trials for this drug/intervention are registered.

### Study Population

**Fig 1:**
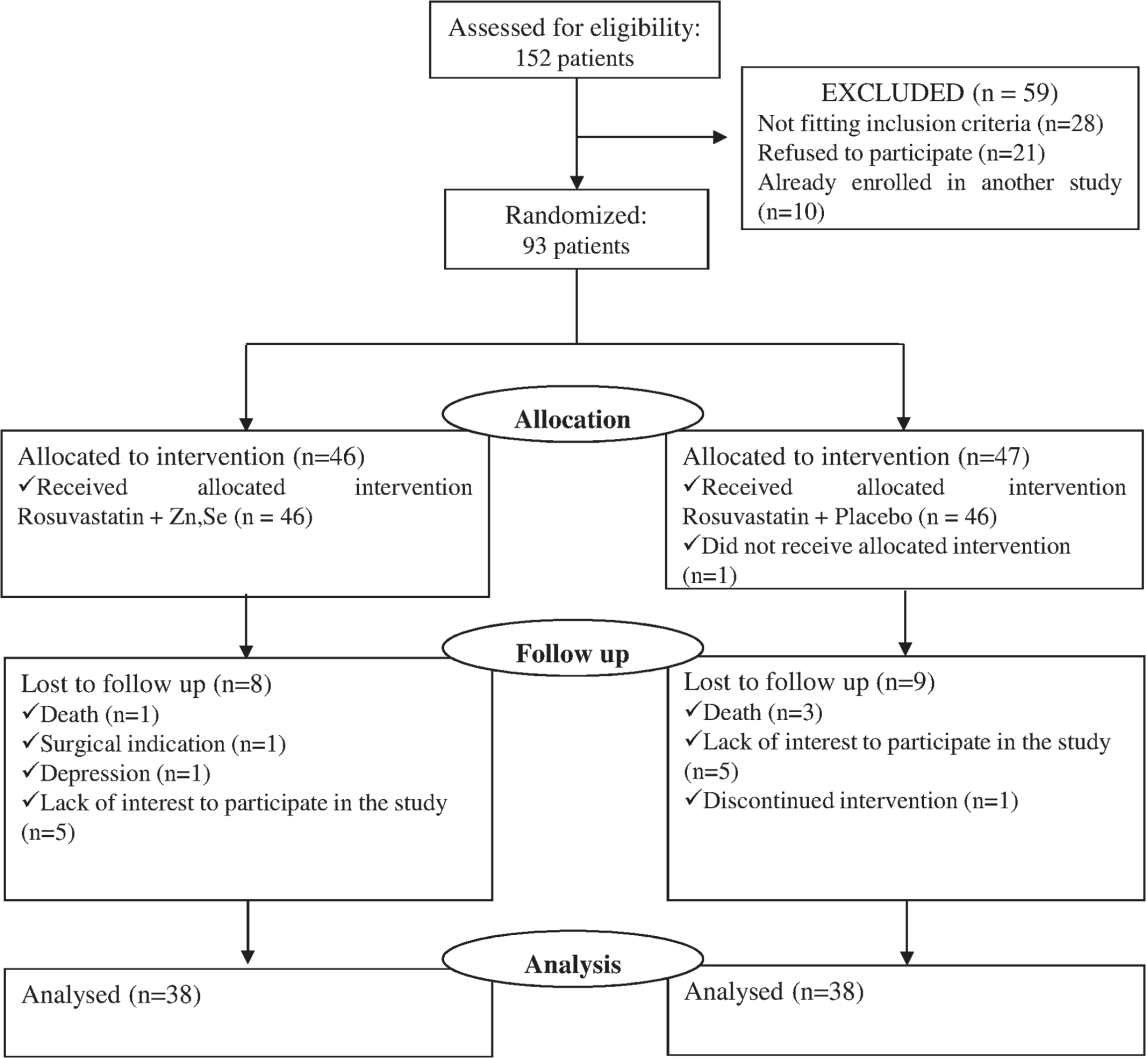
Flow diagram demonstrating participants flow through the study.

In this double-blind randomized clinical trial we evaluated 152 volunteers of both sexes for possible participation in the study. Out of the initial 152 eligible participants identified, 59 were excluded because they did not meet the inclusion criteria (n = 28); refused to participate (n = 21) or they were already enrolled in another study (n = 10). Thus, 93 participants were randomly allocated to receive either rosuvastatin + mineral supplementation (n = 46) or rosuvastatin + placebo (n = 47) (Fig. 1).

All individuals had coronary artery disease and had been submitted to percutaneous coronary angioplasty at the Onofre Lopes Universitary Hospital, Natal, Rio Grande do Norte, Northeast Brazil. Patients were recruited between 01/31/2008 to 12/22/2008 and follow-up between 31/01/2008 to 04/20/2009. To be included in the study, patients needed to have stable angina and meet one of the following criteria: 1) diagnosis of atherosclerosis via angiography revealing >70% stenosis in the lumen in at least 1 segment of a major epicardial artery, or 2) >50% left main coronary artery stenosis. Exclusion criteria included: serious cardiac complications and diseases in the postoperative phase, including thyroid, hematological, congenital, and autoimmune abnormalities, liver disease, kidney failure, neoplasia, and osteoporosis; use of antacids, antibiotics, and/or vitamin-mineral supplements; alcoholism and/or smoking.

### Study Procedures

This study compared the effect of combined oral supplementation with zinc and selenium (30 mg Zn/day + 150 μg Se/day) with the effect of a placebo (inactive pills containing starch identical in appearance to zinc and selenium supplements), both taken in association with 10 mg of rosuvastatin (Crestor^®;^ AstraZeneca, London, UK) over a 4-month period. Zinc and selenium supplements were prepared using Zinc Chelazome^®^ (zinc bis-glycine) and Selenium Complex^®^ (as selenite) by Albion Laboratories, Inc. (Clearfield, UT, USA). We choose the supplementation doses based on known toxicity levels of each mineral, as well as findings indicating that chronically ill patients in Natal, Brazil consume approximately 7 mg/day of zinc and 50 μg/day of selenium (*unpublished data*).

Randomization was performed by the pharmacist at the Companhia da Fórmula (Natal, Brazil), where the supplement capsules containing Zn/Se were produced. Bottles of both supplement and placebo capsules were given unique codes and then delivered to the investigators in batches of 10, with a 50/50 supplement-to-placebo ratio. All information about this process was retained solely by the pharmacist, leaving both study participants and investigators blind to the treatment. Patients were assigned bottles at random, and the code number was recorded so that researchers could later investigate the impacts of each treatment on patient’s lipid profile and antioxidant status. The analyses of the mineral content of the capsules by atomic absorption spectrophotometry showed a zinc content of 26.8 ± 0.6 mg and 155.2 ± 5.5μg of selenium. Zinc and selenium were not detected in the capsules used as placebo.

**Fig 2:**
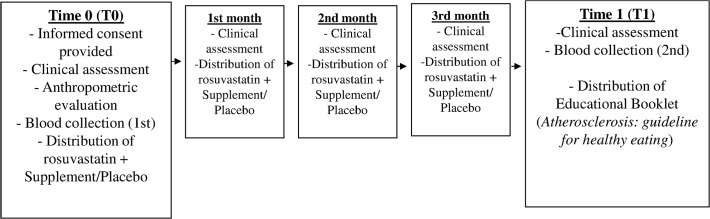
Flow chart describing the monitoring of patients.

Participants attended a preliminary appointment (T0) during which a clinical evaluation was done and an interview was conducted in order to obtain general information for anamnesis. During this baseline visit, we used a 216-cm handheld stadiometer with a platform (WSC^®^, Cardiomed, Curitiba, Brazil) to measure height, and an MEA-03140 solar digital balance (Tanita, Arlington Heights, IL, USA) to measure weight; these values were used to calculate body mass index (BMI). We also measured abdominal circumference (CA) and collected blood in order to perform biochemical evaluations. Four return visits were scheduled at 1-month intervals, for a total of 5 visits per patient. During each monthly visit to the outpatient clinic, patients received both statin medication and a bottle of either mineral supplements or the placebo. At this time, we also performed a clinical assessment, checked for possible side effects, and determined whether patients were adhering to the treatment. In addition to these routine visits, each patient participated in five 24-hour recalls during which we assessed subjects’ dietary zinc and selenium intake. Once the 4-month study period was completed, we again assessed anthropometric data and collected a second blood sample to perform biochemical measurements (Fig. 2).

### Biochemical Assays

Blood samples (30 mL) were obtained after a 12-h fast for biochemical analysis. An AU400 immunochemical analyzer (Olympus, Tokyo, Japan) and Olympus laboratory kits were used to perform analyses of total cholesterol, triglycerides, high density lipoprotein cholesterol (HDL-c), glucose, aspartate aminotransferase (AST), and alanine aminotransferase (ALT). LDL-c was determined using the Friedewald Formula. We measured non-HDL cholesterol (total cholesterol – HDL-c), using the formula provided by the National Cholesterol Education Program (NCEP) III [13]. The serum concentration of hs-CRP was quantified using the CRP-hs^®^ 13927 kit (BioSystems S.A, Costa Brava, Barcelona). Turbidimetric analyses were conducted using the Roche Hitachi-917 automated device (Roche Diagnostics Division,Germany).

An immunosorbent assay was used to measure electronegative low density lipoprotein (LDL(-)) concentrations according to the methods of Faulin et al. [14]. GPx enzyme activity was assessed using a RANSEL^®^ kit (RS504; Randox Laboratory, San Francisco, CA, USA). Ransel Control reference material (SC692; Randox Laboratory) was used to ensure accuracy. Erythrocyte superoxide dismutase activity was measured with a RANSOD^®^ kit (SD125; Randox Laboratory). Ransod Control reference material (SD126; Randox Laboratory) was used to ensure accuracy.

All glassware and plastic containers used during blood collection and mineral analyses were carefully demineralized in nitric acid bath to 30% for at least 12 h, and rinsed using ultra-pure (Milli-Q^®^) after 10 times to minimize minerals contamination. Serum and plasma were separated by centrifugation for 15 min at 3500 rpm at 4°C. The erythrocyte mass obtained was washed three times with 5 mL of 0.9% saline, slowly homogenized by inversion and centrifuged again at 10,000 g for 10 min (SIGMA® 2K15 centrifuge, Germany) at 4°C, after which the supernatant was discarded. Following the final centrifugation, the saline solution was aspirated and the erythrocyte mass was carefully extracted using a micropipette, transferred into demineralized eppendorf tubes and stored for later analysis of zinc and hemoglobin. For assays that were not performed on the day of blood collection, aliquots were stored at −80°C.

Plasma and erythrocyte zinc concentrations were quantified via atomic absorption spectrophotometry using a Spectra Varian AA-240 device (Varian Medical Systems, Inc., Milpitas, CA, USA). Plasma zinc concentrations were determined according to methods that have previously been described elsewhere [15]. Plasma and erythrocyte selenium analyses were performed via atomic absorption spectrophotometry with a Hitachi Z-5000 spectrophotometer (Tokyo, Japan) with hydride generation coupled to a quartz cell; standardization was performed using previously reported techniques [16]. Seronorm™ Trace Elements Serum L-1 (Sero AS, Billingstad, Norway) was used as a reference during zinc and selenium analyses.

### Primary and Secondary Endpoints

The primary endpoint was the reduction in LDL-c levels (in mg/dL) from baseline to 4 months. Secondary endpoints included: changes in total cholesterol, LDL(-), SOD, GPx and zinc and selenium levels.

### Statistical Analysis

The sample size was calculated to detect a difference of at least 30mg/dL in the LDL-c levels assuming a t-test, a population standard deviation of 40 mg/dL and an allocation ratio of 1:1. The approximate population standard deviation of 40mg/dL was based from a previous study, whose population is similar to this studied in the present investigation [17]. We estimated that 38 patients in each arm would be required to achieve a power of 90% at a two-sided significance level of 5%.

Throughout this study, parametric tests were used for approximately normally distributed variables, whereas non-parametric tests were employed when the distribution of the variable was skewed. Data are expressed as means ± standard deviation (SD), median (interquartile range) accordingly. Absolute number (percentage) was used to summarize count data.

The *t*-test for independent samples or the nonparametric Mann-Whitney *U* test were applied to test for between-group differences for baseline characteristics. For intragroup comparisons, we calculated absolute change over time (*Δ*) as: *Δ* = μ_post-treatment −_ μ_pretreatment_, where μ stands for the sample mean. To test the hypothesis that |*Δ*| > 0 (i.e a significant change over time), we used Student’s paired-samples *t*-tests or Wilcoxon’s matched-pairs signed-ranks tests. For binary/categorical variables, we used a Fisher’s exact test, and its extensions for 2×*k* contingency tables. For intergroup comparisons, differences in terms of efficacy (δ) were computed as follows: δ = Δ_supplemented group_ – ∆_placebo group_. For latter analyses, the Student’s *t*-test for independent samples was used. All data analyses were performed using the Stata package (version 8.0, Stata Corp., College Station, TX, USA). Statistical significance was set at the 5% level.

## Results

### Subjects

After 4 months, 38 patients completed the treatment in each group. Reasons for loss of follow up are described in Fig. 1. Composition of the two treatment groups was similar in terms of patient age, sex, body mass index, abdominal circumference, number of atherosclerotic lesions ≥60%, previous coronary or cerebral ischemic events and use of medication prior randomization (Table 1; P > 0.06). Pre-existing hypertension diagnosis was the most common associated disease in both groups, impacting 53% of rosuvastatin + mineral-supplemented individuals, and 74% of rosuvastatin + placebo patients. Both groups had patients with diagnosis of hypertension and type 2 diabetes mellitus (Table 1).

**Table 1:** Baseline characteristics of patients treated with rosuvastatin + mineral supplement or placebo.

	Rosuvastatin + Zn,Se (n = 38)	Rosuvastatin + Placebo (n = 38)	P-value
Age (years)	61.7 ± 9.1	62.8 ± 9.8	0.61
Body mass index (kg/m^2^)	28.3 ± 4.5	27.8 ± 4.6	0.53
Abdominal circumference (cm)	98.8 ± 10.9	97.9 ± 9.9	0.29
Sex (male/female)	24/14	23/15	0.99
Associated Diseases			
None [n (%)]	10 (26)	2 (5)	0.02
Hypertension [n (%)]	20 (53)	28 (74)	
Type 2 diabetes mellitus [n (%)]	2 (5)	0 (0)	
Hypertension + Type 2 diabetes mellitus [n (%)]	6 (16)	8 (21)	
Number of atherosclerotic lesions ≥60%			
1 [n (%)]	20 (53)	18 (47)	0.94
2 [n (%)]	12 (32)	12 (32)	
3 [n (%)]	4 (10)	6 (16)	
≥4 [n (%)]	2 (5)	2 (5)	
Previous coronary or cerebral ischemic events			
None [n (%)]	25 (66)	21 (55)	0.06
Acute myocardial infarction [n (%)]	12 (31)	15 (40)	
Ischemic vascular cerebral accident [n (%)]	1 (3)	2 (5)	
Use of medication prior randomization			
Statins (simvastatin) [n (%)]	19 (50)	13 (34)	0.12
Antiplatelet [n (%)]	38 (100)	38 (100)	
Antihypertensive [n (%)]	38 (100)	38 (100)	

Age, body mass index and abdominal circumference: data are mean ± standard deviation.

The frequency of fasting hyperglycaemia was higher than normal in both groups; however, the intervention was associated with a significant decline in blood glucose values in the rosuvastatin + placebo group (P = 0.0007). The hs-CRP level was significantly reduced in both groups (P<0.001), but without significant differences between the groups (P = 0.21 (Table 2).

**Table 2:** Biochemical biomarkers measured in patients treated with rosuvastatin + mineral supplement or placebo.

	Rosuvastatin + Zn,Se (n = 38)			Rosuvastatin + Placebo (n = 38)			ΔZn,Se) – Δ(Placebo) (n = 76)	
	Baseline	Post	P-value	Baseline	Post	P-value	δ (95% CI)^1^	P-value^2^
Fasting blood glucose (mg/dL)	116.0±32.8	111.3±29.5	0.46	125.0±52.8	112.0±44.5	<0.001	8.4 (-5.6–22.5)	0.24
Total cholesterol (mg/dL)	177 (150–215)	139 (122–159)	<0.001	179 (155–219)	139 (115–174)	<0.001	5.9 (-16.2–27.9)	0.42
LDL-c (mg/dL)	98 (69–132)	64 (49–80.6)	<0.001	106 (84–150)	70 (56–89)	<0.001	6.5 (-13.5–26.5)	0.36
HDL-c (mg/dL)	38 (32–46)	37 (33–45)	0.39	36 (31–44)	36 (32–44)	0.19	-1.1 (-4.8–2.5)	0.59
Triglycerides (mg/dL)	160 (112–276)	129 (103–205)	0.07	169 (116–221)	130 (93–178)	0.03	19.1 (-27.1–65.4)	0.76
Non-HDL-c (mg/dL)	143 (112–176)	102 (77–113)	<0.001	145 (120–176)	100 (78–129)	<0.001	7.0 (-14.3–28.3)	0.31
ALT (U/L)	22 (15–29)	24 (18–32)	0.74	22 (16–30)	24 (17–35)	0.41	-0.1 (-6.2–6.0)	0.97
AST (U/L)	19 (16–25)	20 (17–26)	0.41	18 (16–25)	19 (17–27)	0.87	0.0 (-4.5–4.5)	0.99
hs-CRP (mg/dL)	0.17 (0.07–0.40)	0.04 (0.02–0.14)	<0.001	0.34 (0.08–0.80)	0.11 (0.04–0.29)	<0.001	0.16 (-0.01–0.33)	0.21

Data are mean±standard deviation or median (25–75% interquartile range).

^1^ Difference in response between rosuvastatin + Zn,Se or placebo groups after treatment, with a 95% confidence interval.

^2^ P-values indicate differences between rosuvastatin + Zn,Se or placebo groups after treatment.

Number of participants with missing data: LDL-c (01 rosuvastatin + Zn,Se group; 01 rosuvastatin + placebo group).

ALT = alanine aminotransferase, AST = aspartate aminotransferase, HDL-c = high-density lipoprotein cholesterol, LDL-c = low-density lipoprotein cholesterol, hs-CRP = high sensitivity C-reactive protein, Zn = zinc, Se = selenium.

### Lipid profile

Significant decreases in total cholesterol, LDL-c, and non-HDL-c levels were observed for all patients, regardless of group; however, there were no differences between the rosuvastatin + mineral-supplemented and rosuvastatin + placebo groups (P > 0.21). This confirms the positive effects of rosuvastatin on lipid profile, regardless of mineral supplementation. HDL-c levels were similar in both groups and were consistent across the 4-month study period—likely because the patients neither engage in a physical activity program nor consume foods high in monounsaturated fats. Triglycerides concentrations dropped noticeably in both groups, though this pattern was significant only for the placebo group (P = 0.034); despite this, differences between the two groups were not significant (P = 0.76). Either rosuvastatin + placebo treatment or rosuvastatin + mineral supplemented did not change hepatic enzymes activities (P > 0.41) (Table 2).

### LDL (-), SOD and GPx activities

Neither treatment was associated with statistically significant changes in LDL(-) levels after 4 months (Table 3). Similarly, the activities of the antioxidant enzymes GPx and SOD were unchanged by treatments (P > 0.42) and were similar in both groups (P > 0.33). For all studied patients (regardless of treatment group), the median levels of these enzymes at the end of the 4-month study period were within the reference range (GPx: 28–74 Ug/Hb; SOD: 1102–1601 Ug/Hb) (Table 3).

**Table 3:** LDL(-) levels and SOD and GPx activity measured in patients treated with rosuvastatin + mineral supplement or placebo.

	Rosuvastatin + Zn,Se (n = 38)			Rosuvastatin + Placebo (n = 38)			∆(Zn,Se) – ∆(Placebo) (n = 76)	
	Baseline	Post	P-value	Baseline	Post	P-value	δ (95% CI)^1^	P-value^2^
LDL(-) (U/L)	0.18 (0.10–0.48)	0.17 (0.10–0.55)	0.41	0.48 (0.19–0.91)	0.52 (0.22–0.86)	0.16	−0.04 (−0.16–0.08)	0.52
SOD (U g/Hb)	1397 (1104–1694)	1331 (1112–1613)	0.87	1342 (1222–1681)	1440 (1303–1661)	0.42	−126.3 (−301.0–48.5)	0.33
GPx (U g/Hb)	43 (33–54)	42 (36–56)	0.87	41 (35–48)	44 (36–54)	0.87	−1.0 (−6.0–3.9)	0.68

Data are median (25–75% interquartile range).

^1^ Difference in response between rosuvastatin + Zn,Se or placebo groups after treatment, with a 95% confidence interval.

^2^ P-values indicate differences between rosuvastatin + Zn,Se or placebo groups after treatment.

LDL (-) = Minimally modified electronegative low-density lipoprotein, SOD = superoxide dismutase, GPx = glutathione peroxidase.

### Zinc and selenium status

Neither treatment was associated with significant differences in concentrations of zinc (plasma: P > 0.14; erythrocyte: P > 0.14) or selenium (plasma: P > 0.63; erythrocyte: P > 0.07) of studied groups. Thus, neither rosuvastatin alone nor the rosuvastatin + mineral supplementation influenced the zinc and selenium status (Table 4). The two treatment groups had similar dietary intakes of both zinc (rosuvastatin + mineral-supplemented: 6.97 ± 0.98 mg/day; rosuvastatin + placebo group: 6.94 ± 0.86 mg/day) and selenium (rosuvastatin + mineral-supplemented: 42.19 ± 15.70 μg/day; rosuvastatin + placebo group: 42.56 ± 8.67 μg/day); these values do not reflect the additional 30 mg of zinc and 150 μg of selenium consumed by patients in the supplementation groups.

**Table 4:** Zinc and selenium levels measured in patients treated with rosuvastatin + mineral supplement or placebo.

	Rosuvastatin + Zn,Se (n = 38)			Rosuvastatin + Placebo (n = 38)			∆(Zn,Se) – ∆(Placebo) (n = 76)	
	Baseline	Post	P-value	Baseline	Post	P-value	δ (95% CI)^1^	P-value^2^
Plasma zinc (μg/dL)	84.0 (70.8–101.8)	90.0 (75.4–101.5)	0.14	77.0 (67.3–95.0)	82.4 (72.4–98.2)	0.99	1.4 (-7.7–10.5)	0.77
Erythrocyte zinc (μg/g Hb)	52. 5 (41.1–62.1)	50.8 (41.9–57.5)	0.14	49.2 (42.7–57.9)	50.1 (40.8–59.2)	0.99	-1.8 (-7.0–3.3)	0.19
Plasma selenium (μg/L)	68.0 (59.9–78.8)	70.6 (59.8–76.2)	0.87	63.1(52.1–70.0)	61.9 (56.1–71.6)	0.63	-0.1 (-4.6–4.3)	0.95
Erythrocyte selenium (μg/L)	104.1 (89.2–139)	111.8 (99.2–148)	0.07	106 (89.6–129.8)	114.7 (85–136.6)	0.99	-2.0 (-14.9–10.9)	0.77

Data are median (25–75% interquartile range).

^1^ Difference in response between rosuvastatin + Zn,Se or placebo groups after treatment, with a 95% confidence interval.

^2^ P-values indicate differences between rosuvastatin + Zn,Se or placebo groups after treatment.

Number of participants with missing data: Plasma zinc and Erythrocyte zinc (02 rosuvastatin+ Zn,Se group; 02 rosuvastatin+placebo group).

## Discussion

The 4-month intervention with 10 mg/day of rosuvastatin was associated with a significant decline in total cholesterol, LDL-c, hs-CRP (both groups), blood glucose (rosuvastatin + placebo group only), and triglyceride concentrations (rosuvastatin + placebo group only) without effects on HDL-c levels, hepatic enzymes, or, notably, zinc or selenium status. Likewise, either mineral concentrations or biomarkers of oxidative stress remained stable for both groups across the 4-month treatment, suggesting that neither rosuvastatin alone, nor rosuvastatin in combination with mineral supplements, reduced the biomarker of oxidative stress or improved antioxidant enzymes activity

In our study a relatively low percentage of patients with diagnosis of diabetes was observed in both groups (21%). The impaired fasting glucose may be assigned to poor metabolic control of patients with diabetes, as well as to undiagnosed patients. Abnormal glucose metabolism is substantially more common and usually undiagnosed than previously acknowledged in patients with stable coronary arterial disease [18,19]. Taubert et al (2003) [20] observed that diabetes was previously unrecognized in half of 3266 patients scheduled for coronary angiography.

We have studied the diet of participants in both groups, however, no significant differences were found for all studied nutrients, including those related to glycemic control and triglycerides levels. All patients received the same clinical and nutritional supervision during the four months of study. Although a intra-group difference has been observed for blood glucose (P = 0.0007) and triglycerides (P = 0.003) values for patients treated with rosuvastatin + placebo, no difference for response between treatments over time (P = 0.24 and 0.76, respectively) was found.

Clinical studies have shown positive effects of rosuvastatin on reducing total cholesterol, LDL-c and triglycerides concentrations, and increased HDL-c in patients with dyslipidemia and diabetes mellitus [21,22]. In this study, the positive effects of rosuvastatin treatment on lipid profile were independent of zinc and selenium supplementation, suggesting that either the supplementation conditions used in this protocol were not effective to enhance the pharmacological effect of this statin or that, in fact, zinc and selenium status is not related to this endpoint. In our study, the absence of changes of HDL-c concentration in both groups can be attributed to the fact that most patients did not undertake physical activity besides to have a diet with low intake of foods rich in monounsaturated fat.

Rosuvastatin therapy alone or associated with mineral supplementation did not affect the LDL(-) levels. This may be explained by the fact that this biomarker is higher in individuals with acute coronary syndromes than in those with stable angina [23]. Overall, our results suggest that mineral supplementation may only have small benefits in patients who already have relatively low LDL(-) concentrations and adequate zinc and selenium status. Similarly, Pereira et al. [24]. found that these minerals did not enhance the effects of a combined simvastatin and α-tocopherol treatment, despite the already proven antioxidant effects of zinc and selenium [25–26].

Few studies have assessed the effects of rosuvastatin on the activity of the antioxidant enzymes SOD and GPx-1. Neither of our treatments significantly impacted the activity of these enzymes. The results of this study may not apply to patients with high cardiovascular risk or subjects at an early and pre-clinical phase of coronary arterial disease. Likewise, previous reports found that supplementation with three forms of selenium at three different doses also failed to change patients GPX activity [27]. However, these results contradict a previous report showing that treatment with rosuvastatin (10 mg) by three months can help to protect against oxidative stress by enhancing both GPx and SOD activity [28]. Such conflicting results may stem from differences in analytical techniques, as well as from variations in patient lifestyle and environmental factors; moreover, SOD and GPx activity vary among tissues and organs [29]. It has been suggested that SOD and GPx activities are unaffected by statins because the latter inhibits nicotinamide adenine dinucleotide phosphate-oxidase (NADPH oxidase) activity leading to decreased superoxide generation [30].

Although zinc and selenium are important dietary components that can influence SOD and GPx activity as cofactors of SOD [31] and GPx [32], respectively, no changes in the activities of both enzymes were observed by supplementation with either zinc or selenium in the present study. This is accordingly a previous report showing that a 6-month supplementation with 30 mg/day of zinc gluconate did not change CuZn-SOD activity in diabetic patients [33]. Interestingly, the inadequate dietary zinc intake observed in many patients in our placebo group did not appear to hamper SOD activity—a finding that likely resulted from the presence of strict zinc homeostasis mechanisms [34]. Furthermore, the absence of an effect of zinc supplementation on SOD activity suggests that the amount of zinc administered was not sufficient to compromise copper bioavailability—one potential outcome of zinc supplementation because of the competition between these two minerals in relation to intestinal absorption [35].

Previous reports have suggested that the status of trace elements may be modified by clinical doses of statins [8,10], however our study showed no evidence of this interaction in accordance to data found by Farrokhi et al. [36]. This finding is surprising as statin-induced anti-inflammatory and antioxidant responses, for instance, can be modulated by the activity of metallothioneins, which mediates zinc homeostasis. Zinc itself upregulates genes modulated by statin activity [37]. Moreover, statins interfere with selenoprotein synthesis, an interaction that may be responsible for many of the side effects of these drugs—particularly those observed in patients with myopathies [7].

This is not the first study in which an oral zinc intervention failed to alter zinc status or lead to improvements in oxidative damage and vascular function among supplemented patients [38]. It is possible that, despite a greater demand for zinc, intestinal zinc transporters maintain strict homeostatic control of this mineral [37]. Zinc supplementation appears to be more effective in elderly subjects showing an initial zinc deficiency; study duration and zinc dose are also important elements to consider [39]. Additionally, ingestion of zinc in doses >20 mg may saturate specific zinc pools, leading to a non-linear pharmacokinetics [40]. Indeed, this is responsible for the patterns observed among our supplemented patients.

Bioavailability of selenium can be influenced by a variety of factors including baseline selenium concentrations, interactions between micronutrients in the supplement formulation, prescribed medications, chemical form, dosage, and duration of supplementation [2,41]. Selenite, the chemical form of selenium used in this study, is relatively less effective than organic forms (selenomethionine and selenocysteine) for increasing plasma selenium concentrations. This is because this form enters directly into the selenium pool after it is reduced, whereas selenomethionine is present in the methionine pool until the parent molecule is catabolized [27]. On the other hand, plasma selenium can reach a plateau between the tenth and twelfth week of intervention. Thus, a more detailed analysis of selenium biomarkers would enable a comparison of their relative sensitivities to dietary intake of this mineral. Finally, it would be interesting to examine the possible effects of genotype on selenium metabolism [42].

Several hypotheses have been raised to explain the lack of encouraging results produced by clinical studies involving supplementation with large doses of antioxidants in patients with cardiovascular diseases [43]. Studies may also suffer from biased selection criteria; participants with high baseline levels of increased oxidative stress are more likely than those with lower levels to respond positively to treatment over a shorter period of time [44,45].

Limitations of the present study must to be taken into consideration. Although our study was designed to have 90% power to detect a 30mg/dL mean difference in the LDL-c between groups, our investigation was underpowered to detect more modest changes in the blood lipid profile. In fact, we cannot rule out a smaller effect of the zinc + selenium supplementation on the efficacy of rosuvastatin.

In conclusion, rosuvastatin therapy was efficient in reducing lipids in blood plasma independently of mineral supplementation. Treatment with rosuvastatin did not have significant impacts neither on zinc and selenium status nor on the mineral-associated biomarkers assessed in this study. Further, the zinc and selenium doses used here did not act synergistically or additively with rosuvastatin to modify the lipid profile or SOD and GPx activities of patients with stable angina. Further studies should be focused on testing alternative doses and supplementation time to contribute for a consensus on dose and ideal choice of minerals to be used as possible complementary therapies in atherosclerotic patients.

## Supporting Information

S1 CONSORT Checklist (DOC)

S1 Clinical Trial Register (PDF)

S1 CONSORT 2010 Flow Diagram (DOC)

S1 Protocol Study Protocol in English (DOC)

S2 Protocol Study Protocol in Portuguese. (DOC)
