# Supplementary material for: The Hypolipidemic and Pleiotropic Effects of Rosuvastatin Are Not Enhanced by Its Association with Zinc and Selenium Supplementation in Coronary Artery Disease Patients: A Double Blind Randomized Controlled Study
Source: PLoS One. 2015 Mar 18;10(3):e0119830. doi: 10.1371/journal.pone.0119830 (PMC4365008; doi:10.1371/journal.pone.0119830)
Supplement: S1 Clinical Trial Register — (PDF) [file pone.0119830.s002.pdf]

Protocol Registration Receipt
03/02/2012

Zinc and Selenium Supplementation in Atherosclerosis

This study has been completed.

Sponsor:	Universidade Federal do Rio Grande do Norte
Collaborators:	University of Sao Paulo
Information provided by (Responsible Party):	Karine C M Sena-Evangelista, Universidade Federal do Rio Grande do Norte
ClinicalTrials.gov Identifier:	NCT01547377

Purpose

The aim of this randomized double-blind study was to evaluate the effect of oral zinc and selenium supplementation on oxidative stress and inflammation biomarkers as well as the status of zinc and selenium in patients with atherosclerosis and angina stable treated with rosuvastatin. The hypotheses tested in this study were: Treatment with rosuvastatin impairs zinc and selenium status in patients with atherosclerosis and stable angina? Zinc and selenium supplementation, concomitantly with rosuvastatin, influences the antioxidant and anti-inflammatory as well as the status of minerals?

Condition	Intervention	Phase
Dietary Selenium Deficiency<br>Dietary Zinc Deficiency	Dietary Supplement: zinc and selenium supplementation<br>rosuvastatin + placebo	N/A

Study Type: Interventional
Study Design: Treatment, Parallel Assignment, Double Blind (Subject, Caregiver, Investigator), Randomized, Efficacy Study
Official Title: Effect of Zinc and Selenium Supplementation in Patients With Atherosclerosis Treated With Statins

Further study details as provided by Karine C M Sena-Evangelista, Universidade Federal do Rio Grande do Norte:

Primary Outcome Measure:

- Change from baseline in zinc and selenium status at 4 months [Time Frame: Baseline and 4 months] [Designated as safety issue: Yes]
  We evaluated the effects of 10mg rosuvastatin treatment as well as the effect of treatment with 10 mg rosuvastatin, concomitantly with zinc (30mg/d) and selenium (150µg/d) supplementation on plasma zinc and selenium and on erythrocyte zinc and selenium.

Secondary Outcome Measures:

- Change from baseline in lipid profile at 4 months [Time Frame: Baseline and 4 months] [Designated as safety issue: Yes]
  We evaluated the effects of 10mg rosuvastatin treatment as well as the effect of treatment with 10 mg rosuvastatin, concomitantly with zinc (30mg/d) and selenium (150µg/d) supplementation on total cholesterol, LDL, non-HDL cholesterol and, triglycerides.
- Change from baseline in zinc and selenium status at 4 months [Time Frame: Baseline and 4 months] [Designated as safety issue: Yes]
  We evaluated the effects of 10mg rosuvastatin treatment as well as the effect of treatment with 10 mg rosuvastatin, concomitantly with zinc (30mg/d) and selenium (150µg/d) supplementation on LDL (-), anti-LDL (-), immune complexes concentrations, SOD and GPx activities.
- Change from baseline in inflammation biomarkers status at 4 months [Time Frame: Baseline and 4 months] [Designated as safety issue: Yes]
  We evaluated the effect of oral zinc and selenium supplementation, concomitant with rosuvastatin treatment, on hs-CRP and IL-6 levels.

Enrollment: 76
Study Start Date: January 2008
Study Completion Date: November 2009
Primary Completion Date: April 2009

Arms	Assigned Interventions
Experimental: zinc and selenium supplementation<br>Patients received 10 mg rosuvastatin, concomitantly with zinc (30mg/d) and selenium (150µg/d) supplementation during 4 months	Dietary Supplement: zinc and selenium supplementation<br>Data from patients were obtained at beginning and after four months of treatment with 10 mg rosuvastatin, concomitantly with zinc (30mg/d) and selenium (150µg/d) supplementation or placebo. The anthropometric and dietary data, zinc and selenium concentrations in plasma and erythrocyte, lipid profile, electronegative LDL (LDL(-)), anti- electronegative LDL, Ac-LDL(-) immune complexes, GPx and SOD activities, IL-6 and hs-CRP were evaluated in all patients

Arms	Assigned Interventions
	Other Names:<br>Zinc and selenium supplementation + rosuvastatin
Placebo Comparator: rosuvastatin + placebo<br>Patients received 10 mg rosuvastatin concomitantly placebo pills similar zinc and selenium supplementation	rosuvastatin + placebo<br>Data from patients were obtained at beginning and after four months of treatment with 10 mg rosuvastatin, concomitantly with zinc (30mg/d) and selenium (150µg/d) supplementation or placebo. The anthropometric and dietary data, zinc and selenium concentrations in plasma and erythrocyte, lipid profile, electronegative LDL (LDL(-)), anti- electronegative LDL, Ac-LDL(-) immune complexes, GPx and SOD activities, IL-6 and hs-CRP were evaluated in all patients<br>Other Names:<br>placebo group

The study included 47 men and 29 women, average age around 60 years, with coronary atherosclerosis diagnosed by angiography. Data from patients were obtained at beginning and after four months of treatment with 10 mg rosuvastatin, concomitantly with zinc (30mg/d) and selenium (150µg/d) supplementation or placebo. The anthropometric and dietary data, zinc and selenium concentrations in plasma and erythrocyte, lipid profile, electronegative LDL (LDL(-)), anti- electronegative LDL, Ac-LDL(-) immune complexes, GPx and SOD activities, IL-6 and hs-CRP were evaluated in all patients.

Eligibility

Ages Eligible for Study: 41 Years to 80 Years
Genders Eligible for Study: Both

Inclusion Criteria:

- The study included adult and elderly patients, with coronary atherosclerosis and stable angina diagnosed by angiography showing ≥ 70% stenosis of the vessel lumen in at least one segment of a major epicardial artery or ≥ 50% stenosis of the diameter of the left main coronary artery, stable angina

Exclusion Criteria:

- Cardiac complications or other serious diseases such as:
  - thyroid,
  - hematologic,
  - congenital,
- autoimmune liver disease,
- kidney failure,
- cancer,

- associated infections,
- osteoporosis,
- post-operative,
- use of:
    - antacids,
    - antibiotics and
    - vitamin-mineral supplements,
- alcohol and
- current smoking.

Contacts and Locations

Locations

Brazil

Onofre Lopes University Hospital
Natal, Rio Grande do Norte, Brazil, 59.012.300

Investigators

Study Director:	Dulcineia SP Abdalla, PhD	University of São Paulo
Study Director:	Lucia FC Pedrosa, PhD	University of Rio Grande do Norte

More Information

Responsible Party:	Karine C M Sena-Evangelista, Principal Investigator, Universidade Federal do Rio Grande do Norte
Study ID Numbers:	PROJ-981-199697778
Health Authority:	Brazil: Ethics Committee
