# Supplementary material for: The Hypolipidemic and Pleiotropic Effects of Rosuvastatin Are Not Enhanced by Its Association with Zinc and Selenium Supplementation in Coronary Artery Disease Patients: A Double Blind Randomized Controlled Study
Source: PLoS One. 2015 Mar 18;10(3):e0119830. doi: 10.1371/journal.pone.0119830 (PMC4365008; doi:10.1371/journal.pone.0119830)
Supplement: S1 CONSORT 2010 Flow Diagram — (DOC) [file pone.0119830.s003.doc]

**CONSORT 2010 Flow Diagram**

**Allocation**

**Analysis**

**Follow-Up**

**Enrollment**

Assessed for eligibility (n= 152)

Excluded (n= 59)

♦  Not fitting inclusion criteria (n= 28)

♦  Refuse to participate (n= 21)

♦  Other reasons (n= 10)

Analysed (n=38)

**Lost to follow-up (n=8)**

Death (n=1), Sirurgical indication (n=1), Depression (n=1), lack of interest to participate in the study (n=5)

**Allocated to intervention mineral-supplementation (n= 46)**

♦ Received allocated intervention (n= 46)

**Lost to follow-up (n=9)**

Death (n=3), lack of interest to participate in the study (n=5), discontinued intervention (n=1)

**Allocated to placebo (n=47)**

♦ Received allocated intervention (n= 46)

♦ Did not receive allocated intervention (n=1)

Analysed (n=38)

Randomized (n= 93)
