# Supplementary material for: The Hypolipidemic and Pleiotropic Effects of Rosuvastatin Are Not Enhanced by Its Association with Zinc and Selenium Supplementation in Coronary Artery Disease Patients: A Double Blind Randomized Controlled Study
Source: PLoS One. 2015 Mar 18;10(3):e0119830. doi: 10.1371/journal.pone.0119830 (PMC4365008; doi:10.1371/journal.pone.0119830)
Supplement: S1 Protocol — (DOC) [file pone.0119830.s004.doc]

SAO PAULO UNIVERSITY

FACULTY OF PHARMACEUTICAL SCIENCES

FOOD SCIENCE AND NUTRITION GRADUATE PROGRAM

STUDY PROTOCOL

**EFFECT OF SUPPLEMENTATION WITH ANTIOXIDANT MINERALS ON PATIENTS WITH STATIN-TREATED ARTHEROSCLEROSIS**

INVESTIGATORS

KARINE CAVALCANTI MAURÍCIO DE SENA

Dulcineia Saes Parra Abdalla

Sao Paulo

2006

**1. INTRODUCTION AND JUSTIFICATION**

Atherosclerosis is characterized by the accumulation of cholesterol deposits in the macrophages present in medium and large arteries. This deposition process favors proliferation of certain types of cells on the arterial wall that invade the lumen of the vessel, compromising blood flow. The clinical forms of atherosclerosis are often referred to as coronary artery disease or cerebrovascular disease, which result in functional alterations of the heart and brain, caused by reduced blood flow ^(1,2)^.

There are several hypotheses to explain the processes associated with the development of atherosclerosis. The response-to-injury hypothesis indicates that a vascular lesion is the initial event in the atherosclerosis process. By contrast, the response-to-retention theory places the interactions between lipoproteins and the matrix as the critical point of atherosclerosis, while the hypothesis of oxidative modification highlights the importance of LDL oxidation as the primary triggering factor of the disease^(1)^.

In this respect, the inflammatory response, vascular endothelial dysfunction and oxidative stress are important factors linked to atherogenesis, intermediating the relationship between risk factors and the induction of atheromatous plaque formation. Inflammation, in particular, is the basic phenomenon that determines plaque instability, by means of inflammatory events such as mononuclear leukocyte infiltration and increase in the expression and activity of matrix metalloproteinase, as well as increase in the expression of prothrombotic and apoptosis factors of smooth and endothelial muscle cells. These processes may be favored by oxidative stress ^(3)^.

On the other hand, many antioxidant substances can prevent or delay the oxidation of substrates involved in oxidative stress reactions. With respect to atherosclerosis, antioxidants need to protect vessels against the action of reactive oxygen and nitrogen species, in and outside the cell. Enzymatic antioxidants, mainly superoxide dismutase (SOD) (Cu-ZnSOD, MnSOD), catalase, glutathione peroxidase, glutathione reductase and transferases, thiol-disulfide oxidoreductase and peroxiredoxins are present in arterial wall cells ^(1)^.

In this perspective, Fields (1999)^(4)^ showed the relationship between mineral disturbances and the atherosclerotic process. High concentrations of iron and copper can have a direct effect on the LDL oxidation process. Even though copper acts as a pre-oxidizing substance at high concentrations, it is also a component of Cu-ZnSOD and ceruloplasmin. Therefore, its excess or deficiency may favor LDL oxidation, hypercholesterolemia and coronary disease^(4)^.

In relation to zinc and selenium, Henning et al (1999)^(5)^ and Alarcón-Navarro and Martinez (2000)^(6)^ reported that these minerals are important for maintaining endothelial integrity and act as an antiatherogenic substance, inhibiting events related to oxidative stress. Other authors have recently reported the protective effect of zinc in the vascular endothelium, owing to its antioxidant and cell membrane stabilizing role, in addition to its participation in NF-kB, caspase enzyme and nitric oxide synthase activity ^(7,10)^. Selenium, on the other hand, can also decrease the NF-kB response to the pro-inflammatory mechanism of endothelial cells, limiting the emergence of atherosclerosis ^(8)^.

Zinc and methallotionein deficiency, in particular, can increase the processes related to oxidative stress in endothelial cells, and since this alteration in available plasma zinc affects endothelial zinc status, the deficiency of this mineral may be a risk factor for the formation of atherosclerotic plaque ^(9)^. Selenium deficiency, in turn, compromises the action of glutathione peroxidase and thioredoxin reductase, with the consequent accumulation of hydroperoxides that may induce LDL oxidation ^(7)^.

In regard to the treatment of patients with atherosclerosis, there is a need for a multifactorial and simultaneous approach in relation to all the risk factors, initially prioritizing changes in lifestyles, including nutritional therapy, physical activity, and tobacco abstinence, associated or not to specific pharmacological treatment. For the last one, the use of HMG-CoA reductase inhibitors is recommended for exerting effects on the vasomotor function of the endothelium, inflammation and thrombosis, in addition to inhibiting cholesterol biosynthesis. In this regard, statins are the drugs of choice (lovastatin, fluvastatin, atorvastatin, simvastatin, pravastatin, and rosuvastatin) ^(11)^.

These drugs, in addition to decreasing serum cholesterol levels, exhibit a series of vascular protection or “pleiotropic” actions that include enhanced endothelial function, increased NO bioavailability, atherosclerotic plaque stabilization, inflammatory response inhibition and immunomodulatory actions, thereby reducing the incidence of heart attack in patients treated with HMG-CoA reductase inhibitors ^(12)^.

Considering the importance of metals (ex.: iron, copper, zinc, selenium) in oxidative processes, a number of studies show the relationship between these elements and the use of statins. Leonhardt et al (1997)^(13)^ concluded that treating hypercholesterolemic patients with fluvastatin for 8 weeks resulted in lower total cholesterol, triglycerides, and plasma zinc, underscoring the importance of the last, given that zinc is directly related to CuZnSOD dismutase activity.

Corroborating these results, Ghayour-Mobarhan et al (2005)^(14)^ assessed the effect of therapy with simvastatin and atorvastatin for 4 months in dyslipidemic patients (case-control study), observing a significant post-treatment decrease in total cholesterol, LDL-cholesterol, serum zinc, copper, ceruloplasmin and reactive C protein; with no alteration in serum selenium, the copper/ceruloplasmin ratio and glutathione peroxidase in the statin group. The study demonstrates that the levels of these trace minerals may be significantly affected by treatment with statin doses routinely used for clinical treatment.

Moosmann and Behl (2004)^(15)^ emphasize that some of the side effects of statins, such as myopathy, rhabdomyolysis and polyneuropathy, are similar to those observed in selenium deficiency. The authors suggest that the negative effect of statins on the synthesis of selenoproteins seems to explain some of the side effects of statins, especially myopathy.

Accordingly, the present study aims at assessing the effect of supplementation with oxidizing minerals, zinc and selenium, in patients with coronary heart disease treated with statins, identifying the potential effect of these minerals with the simultaneous use of statins on markers of oxidative stress, inflammation and antioxidizing enzymes.

**2. OBJECTIVES**

**2.1 General objective:**

- To evaluate the effect of oral zinc and selenium supplementation on oxidative stress in patients with coronary heart disease (CHD) exhibiting stable angina and treated with statins.

**2.2 Specific objectives:**

- To evaluate the anthropometric nutritional status and dietary intake of patients;

- To identify the nutritional status of patients with respect to zinc and selenium before and after supplementation;

- To determine the lipid profile, oxidized LDL, inflammation and antioxidant enzyme markers in the studied groups before and after zinc and selenium supplementation;

- To correlate zinc and selenium concentrations with lipid profile, oxLDL, and markers of inflammation and antioxidant enzymes in the studied groups.

**3. WORK PLAN – EXECUTION SCHEDULE**

| Aug | Sep | Oct | Nov | Dec |  |
| --- | --- | --- | --- | --- | --- |
|  |  |  |  |  | **Submission of the Project to the Ethics Committee** |
|  |  |  |  |  | **Material Acquisition** |
|  |  |  |  |  | **Standardization of Methodologies**  * Assessment and data collection for:  Anthropometric and dietary analyses  Lipid profile analysis  Biochemical analysis of zinc and selenium  Analysis of oxidative stress and inflammatory markers  Analysis of antioxidizing enzymes |

| Jan | Feb | Mar | Apr | May | Jun | Jul | Aug | Sep | Oct | Nov | Dec |  |
| --- | --- | --- | --- | --- | --- | --- | --- | --- | --- | --- | --- | --- |
|  |  |  |  |  |  | **Standardization of Methodologies** | | | | | |  |
|  |  |  |  |  |  | **Preparation /acquisition of supplementation and medication** | | | | | |  |
|  |  |  |  |  |  |  | **Contacts with the ambulatory facility to initiate collections** | | | | |  |
|  |  |  |  |  |  |  |  |  |  |  |  | **Participant selection**  **1^st^ assessment (Data collection)** |
|  |  |  |  |  |  |  |  |  |  |  |  |  |
|  |  |  |  |  |  |  |  |  |  |  |  | **Onset of supplementation** |

| Jan | Feb | Mar | Apr | May | Jun | Jul | Aug | Sep | Oct | Nov | Dec |  |
| --- | --- | --- | --- | --- | --- | --- | --- | --- | --- | --- | --- | --- |
|  |  |  |  |  |  | **2^nd^ assessment (data collection) post-supplementation** | | | | | |  |
|  |  |  |  |  |  |  | | | | | |  |
|  |  |  |  |  |  |  |  |  |  |  |  | **Biochemical Analyses**  **Dietary analyses** |

| Jan | Feb | Mar | Apr | May | Jun | Jul | Aug | Sep | Oct | Nov | Dec |  |
| --- | --- | --- | --- | --- | --- | --- | --- | --- | --- | --- | --- | --- |
|  |  |  | **Unblinding of the study – statistical analyses**  **Data tabulation**  **Qualification** | | | | | | | | |  |
|  |  |  |  |  |  |  |  | **Preparation of the final study, thesis defense and writing the article** | | | |  |

**4. MATERIAL AND METHODS**

**4.1. Experimental Protocol**

The is a double-blind randomized trial that will be conducted with a group of adults and elderly patients with clinical diagnosis of coronary heart disease submitted to angioplasty, treated at the Hemodynamic Center of Onofre Lopes University Hospital (HUOL) at the Rio Grande do Norte Federal University (UFRN), Natal, Brazil between August 2007 and June 2008 (Flowchart 1).

Patients were recruited at the Ambulatory Facility of the aforementioned institution, screened by the researcher and doctor in charge, considering the following inclusion criteria: adult and elderly patients of both sexes diagnosed with coronary artery disease by angiography with stenosis > 70% in one or more arteries. Exclusion criteria were the presence of severe cardiac complications or other disorders such as hematologic or autoimmune diseases, hepatopathies, kidney failure, neoplasias, diabetes mellitus, postoperative infections, use of antacids, antibiotics and/or vitamin/mineral supplements, alcoholism and smoking.

Sample size ^(16)^ was calculated assuming 90% statistical power to detect a difference of at least 0.075nmol/mg between measures of oxidized LDL in the experimental and control group. A difference of 0.075nmol/mg was considered clinically significant based on previous studies^(17)^. Parameters such as mean and population variance were based on studies with large samples^(17,18)^ that assessed Brazilian populations similar to the groups to be studied. The simulations revealed a sample size of 76 individuals (38 experimental and 38 controls) in order to detect a 90% statistical power and a significance level of 0.05.

Patients with atherosclerosis that formed the groups will be selected by random draw. The experimental group will be composed of patients with atherosclerosis who will be submitted to treatment with statin + zinc and selenium supplementation and the control group consisting of patients who received statin + placebo.

Patients from the atherosclerosis group will be treated with rosuvastatin from the Astra|Zeneca® laboratory at a dose of 10 mg/day. Zinc and selenium supplements will be prepared from Taste Free® zinc amino acid and Selenium Complex® from Albion Laboratories Incorporation and the drug formula prepared according to manufacturer’s recommendations.

Mineral levels will be determined considering DRI recommendations (2001), which define a maximum daily zinc and selenium intake for adults of both sexes aged 50 years or older of 40mg/day and 400µg/day, respectively. Therefore, considering the results of earlier studies assessing zinc and selenium intake in patients with non-transmissible chronic diseases treated at an ambulatory facility in Natal, which revealed a mean intake of 7mg/day of zinc and 50µg/day of selenium, the doses used will be 30mg/Zn/day and 150µgSe/day. Patients will be accompanied monthly at the Ambulatory facility in order to monitor use of the drug+supplement, clarify doubts and identify complications or adverse reactions.

Data collection will be conducted at the onset and after 4 months of treatment and will include the following parameters: assessment of nutritional status in relation to zinc and selenium, selenoprotein P, lipid profile, minimally oxidized LDL (electronegative LDL), markers of inflammation and antioxidizing enzymes. Anthropometric and dietary assessment will be carried out to characterize the population in terms of anthropometric and dietary nutritional status.

**4.1.1 Ethical Aspects**

The study will be conducted in accordance with the regulatory guidelines of studies involving human beings, as stated in Resolution 196/96 of the National Health Council (Ministry of Health, 1996) and will be analyzed by the Research Ethics Committee of Onofre Lopes University Hospital (HUOL) at UFRN and the Faculty of Pharmaceutical Sciences at Sao Paulo University (USP). Data will only be collected after patients give their informed consent and are advised of the aims, risks and benefits of the research. The study will be conclude at the end of the steps outlined in the schedule of activities.

Participants will be informed about the formation of the experimental and control groups (placebo), and can withdraw their consent at any moment and leave the study without compromising their follow-up. Study participation will be free of charge to the patients, who will not be remunerated except for transportation and food expenses. The data obtained from this research will only be used for scientific purposes and both the image and identity of the participant will be preserved.

The possible risks of taking part in this investigation are related to blood collection, such as bleeding or slight bruising. Thus, blood collections will take place at the laboratory of Onofre Lopes University Hospital by trained professionals and any complication will be referred to the first-aid sector of the hospital. According to manufacturer’s information, the medication (rosuvastatin) prescribed by the medical doctor may cause the following reactions: - **Rare:** unpleasant muscle effects and severe allergic reactions, that may affect respiratory pathways; - **Uncommon:** slight reactions that disappear rapidly such as itching, redness and allergic skin reactions; - **Common:** headache, muscle pain, stomach’s ache, general feeling of weakness, constipation, dizziness and nausea. The use of zinc and selenium supplements at recommended doses showed no side effects. Patients will be instructed to inform the doctor and/or researcher of any complications so that the appropriate measures can be taken.

The results of this study will reveal the anthropometric measures of nutritional and dietary status of patients with atherosclerosis, as well as metabolic alterations of zinc and selenium in patients using rosuvastatin, considering the important role of these minerals in the antioxidant defense and inflammatory mechanisms, besides to support more specific medical-nutritional conduct that can be directed to treat this group of patients.

**FLOWCHART 1**

**DOUBLE-BLIND RANDOMIZED CLINICAL TRIAL**

**Sample**

Patients with atherosclerosis

Hemodynamic sector of HUOL/UFRN

- Obtain informed consent

- Anthropometric assessment

- Hand out the 3-day food record form

- Schedule blood collections and return of the Food Record form.

**Random Draw**

**Group 1**

Statin + Zn and Se Sup.

**Group 2**

Statin + Placebo

Lipid/

ALT/ AST profile

Ultrasensitive PCR

Plasma/Zinc erythrocytes

Selenium Plasma/

erythrocytes

Selenoprotein P

CuZnSOD

GPx

LDL-

E-selectin

**Assessments**

**T1 and T2**

**4-month segment**

IL-8

Clinical Analyses Lab.– HUOL-UFRN

UFRN

Mult, Lab

UFRN

Lab Nut. and B14 Minerals - USP

Clinical Biochemical Laboratory – B 17 - USP

**4.2. Anthropometric Assessment**

Height and weight will be measured using a 216-cm handheld stadiometer with a platform (WSC®, Cardiomed, Curitiba, Brazil), and an MEA-03140 solar digital balance (Tanita, Arlington Heights, IL, USA), respectively. These values will be used to calculate body mass index (BMI) according to the WHO (2004)^(19)^. We also measured abdominal circumference (CA) according to the WHO (2004)^(19)^.

**4.3. Dietary Assessment**

The 3-day dietary recall will be used to record all food items consumed by patients and controls for 3 consecutive days (2 weekdays and 1 weekend day), applied at the start of the study. The data collected will be analyzed by specific diet analysis programs and recommended energy, macronutrient, fiber, zinc and selenium levels will be assessed in accordance with Brazilian Cardiology Society guidelines (2001)^(20)^ and DRI 2000^(21)^ and 2001^(22)^.

**4.4. Collection of Biological Material**

30mL blood samples will be collected with disposable material, in the morning, after a 12-14 hour fast. Samples will then be distributed into tubes, with or without anticoagulants, submitted to specific procedures for each analysis and stored at a temperature of -80^o^C for subsequent analyses, as follows:

| **TUBE** | **SAMPLE** | **ANTICOAGULANT** | **ANALYSES** |
| --- | --- | --- | --- |
| 1 | 6 mL | Without Anticoagulant | Lipid Profile, AST, ALT  Ultrasensitive PCR |
| 2 | 6 mL | 30% Sodium citrate | Plasma Zinc, Erythrocyte Zinc  Erythrocyte Hemoglobin |
| 3 | 10mL | EDTA | Plasma Selenium , Erythrocyte Selenium, LDL -, selenoprotein P |
| 4 | 8 mL | Heparin | CuZnSOD, GPx,  E-Selectin and IL-8 |

**4.5. Biochemical Analyses**

Analyses will be performed at the Clinical Analysis Laboratory of HUOL/UFRN, Multidisciplinary Research Laboratory, Department of Pharmacy, UFRN, Laboratory of Nutrition and Minerals and Clinical Biochemistry, Faculty of Pharmaceutical Sciences, Sao Paulo University.

**4.5.1. Assessment of Lipid Profile and Liver Function**

An AU400 immunochemical analyzer (Olympus, Tokyo, Japan) and Olympus laboratory kits will be used to analyze total cholesterol, triglycerides, high-density lipoprotein (HDL) cholesterol, glucose, aspartate aminotransferase (AST), alanine aminotransferase (ALT). and low-density lipoprotein (LDL) cholesterol will be determined using the Friedewald Formula. We will measure non-HDL cholesterol (total cholesterol – HDL) using the formula provided by the National Cholesterol Education Program (NCEP) III ^(23)^.

**4.5.2. Biochemical assessment of zinc and selenium**

All glassware and plastic containers used during blood collection and mineral analyses will be carefully demineralized in nitric acid bath to 30% for at least 12 h, and rinsed 10 times using ultra-pure (Milli-Q®) to minimize mineral contamination. Serum and plasma will be separated by centrifugation for 15 min at 3500 rpm at 4 ◦C. The erythrocyte mass obtained will be washed three times with 5 mL of 0.9% saline, slowly homogenized by inversion and centrifuged again at 10,000 g for 10 min (SIGMA® 2K15 centrifuge, Germany) at 4 ◦C, after which the supernatant will be discarded. Following the final centrifugation, the saline solution will be aspirated and the erythrocyte mass carefully extracted using a micropipette, transferred into demineralized Eppendorf tubes and stored for subsequent analysis of zinc and hemoglobin. For assays that will be not performed on the day of blood collection, aliquots will be stored at −80 ◦C.

Plasma and erythrocyte zinc concentrations will be quantified via atomic absorption spectrophotometry using a Spectra Varian AA-240 device (Varian Medical Systems, Inc., Milpitas, CA, USA). Plasma zinc concentrations will be determined according to methods described elsewhere^(24)^. Plasma and erythrocyte selenium analyses will be performed by atomic absorption spectrophotometry using a Hitachi Z-5000 spectrophotometer (Tokyo, Japan) with hydride generation coupled to a quartz cell; standardization will be performed using previously reported techniques^(25)^. Seronorm™ Trace Elements Serum L-1 (Sero AS, Billingstad, Norway) will be used as a reference during zinc and selenium analyses.

**4.5.3. Determination of selenoprotein P**

Determination of selenoprotein P will be conducted applying the ELISA assay, using anti-selenoprotein P monoclonal antibody (LF-MA0141) from the LabFrontier® laboratory.

**4.5.4. Assessment of oxidized LDL (LDL^-^)**

An immunosorbent assay will be used to measure LDL(-) concentrations according to methods proposed by Faulin et al.^(26)^.

**4.5.5. Assessment of Glutathione Peroxidase and CuZnSOD dismutase**

GPx enzyme activity will be assessed using a RANSEL® kit (RS504; Randox Laboratory, San Francisco, CA, USA). Ransel Control reference material (SC692; Randox Laboratory) will be used to ensure accuracy. Erythrocyte superoxide dismutase activity will be measured with a RANSOD® kit (SD125; Randox Laboratory). Ransod Control reference material (SD126; Randox Laboratory) will be used to ensure accuracy.

**4.5.6. Assessment of inflammatory markers**

**4.5.6.1. Determination of C-Reactive Proteins (CRP)**

CRP concentrations will be quantified in serum at ultrasensitive levels using the Biotechnical kit (CAT BT-20.017.00)®, following guidelines proposed in the kit, and data will be presented in mg/dL.

**4.5.6.2. Determination of E-selectin**

The plasma level of E-selectin will be determined using the R&D Systems(BBE 2B)®  Human E-selectin Immunoassay kit, in accordance with the manufacturer’s protocol. Readings will be conducted at 450nm in a microplate luminometer and the values will be presented in ng/mL.

**4.5.6.3. Determination of IL-8**

Determination of IL-8 will be made using R&D Systems(D8000C)® Human kit CXCL8/IL-8, considering kit instructions, and readings were performed at 450nm in the microplate luminometer. Data will be expressed in pg/mL.

**5. STATISTICS ANALYSIS**

Data are expressed as mean ± standard deviation (SD), median (interquartile range), or total count (percentage), as appropriate. For intragroup comparisons, data will be calculated in absolute change over time (*∆*) as: *∆* = μ_post-treatment −_ μ_pretreatment_, where μ stands for the sample mean. Student’s paired-samples *t*-tests and Wilcoxon’s matched-pairs signed-ranks tests will be applied to detect statistically significant changes over time. Due to low sample sizes for some categorical or binary variables, Fisher’s exact test and its extensions for 2×*k* contingency tables will be used to compare count data. For intergroup comparisons, differences (δ) will be computed as follow: δ = ∆_supplemented group_ – ∆_placebo group_. For these analyses, the Student’s *t*-test for independent samples will be used for normally distributed variables, whereas the Mann-Whitney *U*-test will be applied for variables with asymmetric distribution. Estimates of δ will be adjusted for potential confounding variables, and calculated by multiple linear regression models.

FINANCING:

This Project will be submitted to research funding agencies, such as the National Research Council (CNPq) or The State of São Paulo Research Foundation (FAPESP) for financing. Funds from the Laboratories where analyses will be carried out will also be sought.

REFERENCES

1. STOCKER, R., KEANEY, J.F. Role of oxidative modifications in atherosclerosis. **Physiol Rev**, v. 84, p.1381-1478, 2004.

2. SCHOEN, F.J. **Os vasos sangüíneos**. In: KUMAR.V, ABBAS A.K., FAUSTO, N. Robbins e Cotran: Patologia – bases patológicas das doenças. Rio de Janeiro:Elsevier. 2005, cap.11, 537-581p.

3. SOUZA, H.P.; LAURINDO, F.R.M. Estresse oxidativo e ruptura da placa aterosclerótica. **Rev Soc Cardiol Estado de São Paulo**, v.12(4), p.584-594, 2002.

4. FIELDS, M. Role os trace elements in coronary heart disease. **British J Nutr**, v. 81, p.85-86, 1999.

5. HENNING, B., MEERARANI, P.; TOBOREK, M.; McCLAIN, C.J. Antioxidant-like properties of zinc in activated endothelial cells. **J Am Coll Nutr**, v.18(2), p.152-158, 1999.

6. ALARCÓN-NAVARRO, M., MARTINEZ, M.C.L. Essentiality of selenium in the human body: relationship with different disease. **Sci Total Environ,** v. 249, p.347-371, 2000.

7. BEATTIE, J.H.; KWUN, I.S. Is zinc deficiency a risk factor for atherosclerosis. **British J Nutr,** v. 91, p.177-181, 2004.

8. [Zhang F](http://www.ncbi.nlm.nih.gov/entrez/query.fcgi?db=pubmed&cmd=Search&itool=pubmed_Abstract&term="Zhang+F"%5BAuthor%5D), [Yu W](http://www.ncbi.nlm.nih.gov/entrez/query.fcgi?db=pubmed&cmd=Search&itool=pubmed_Abstract&term="Yu+W"%5BAuthor%5D), [Hargrove JL](http://www.ncbi.nlm.nih.gov/entrez/query.fcgi?db=pubmed&cmd=Search&itool=pubmed_Abstract&term="Hargrove+JL"%5BAuthor%5D), [Greenspan P](http://www.ncbi.nlm.nih.gov/entrez/query.fcgi?db=pubmed&cmd=Search&itool=pubmed_Abstract&term="Greenspan+P"%5BAuthor%5D), [Dean RG](http://www.ncbi.nlm.nih.gov/entrez/query.fcgi?db=pubmed&cmd=Search&itool=pubmed_Abstract&term="Dean+RG"%5BAuthor%5D), [Taylor EW](http://www.ncbi.nlm.nih.gov/entrez/query.fcgi?db=pubmed&cmd=Search&itool=pubmed_Abstract&term="Taylor+EW"%5BAuthor%5D), [Hartle DK](http://www.ncbi.nlm.nih.gov/entrez/query.fcgi?db=pubmed&cmd=Search&itool=pubmed_Abstract&term="Hartle+DK"%5BAuthor%5D). Inhibition of TNF-alpha induced ICAM-1, VCAM-1 and E-selectin expression by selenium. **Atherosclerosis**, v.161(2), p. 381-386, 2002.

9. HENNING, B., MEERARANI, P.; RAMADASS, P.; TOBOREK, M.; MALECKI, A.; SLIM, R.; McCLAIN, C.J. Zinc nutrition and apoptosis of vascular endothelial cells: implications in atherosclerosis. **Nutrition**, v.15, p. 744-748, 1999.

10. ALISSA, E.M; BAHJRI, S.M.; AHMED, W.H.; AL-AMA, N.; FERNS, G.A.A. Trace elements status in Saudi patients with established atherosclerosis. **J Trace Elem Med Biol**, in press, 2006.

11. KINLAY, S. Potential vascular benefits of statins. **Am J Med**, v. 118(12A), p.625-675, 2005.

12. ENDRES, M. Statins: Potential and indications in inflammatory conditions. **Atherosclerosis** Supplements., v.7, p.31-35, 2006.

13. LEONHARDT, W.; KURKTSCHIEV, W.L.; MEISSNER, D.; LATTKE, P; ABLETSHAUSER, C.; WEIDINGER, G.; JAROSS, W.; HANEFELD, M. Effects os fluvastatin therapy on lipids, antioxidants, oxidation of low density lipoproteins and trace metals. **Eur J Clin Pharmacol**, v. 53, p.65-69, 1997.

14. GHAYOUR-MORBARHAN,M.; LAMB, D.J.; TAYLOR, A.; VAIDYA, N.; LIVINGSTONE, C.; WANG, T; FERNS, G.A.A. Effect of statin therapy on serum trace element status in dyslipidemic subjects. **J Trace Elem Med Biol**, v.19, p.61-67, 2005.

15. MOOSMAN, B.; BEHL,C. Selenoprotein synthesis and side-effects of statins. **Lancet**, v. 363, p.892-894, 2004.

16. GOTTLEIB, M.G; SCHWANKE, C.H.; SANTOS, A.F; JOBIM, P.F.; MUSSEL, D.P.; CRUZ,I.B. Association among oxidized LDL levels, MnSOD, apolipoprotein and polymorphisms, and cardiovascular risk factors in a south Brazilian region population. **Genet Mol Res**, v.4(4), p. 691-703, 2005.

17. LUZ, P.L.; CESERA, F.H; FAVARATO, D. CERQUEIRA, E.S.Comparasion of serum lipids values in patients with coronary artery disease at <50, 50 to 59, 60 to 69, and >70 years of age. **Am J Cardiol**, v. 96(12), p. 1640-1643, 2005.

18. ARAÚJO, F., PEREIRA, A.C., LATORRE, M.R., KRIEGER, J.E., MANSUR, A.J. High-sensitivity C reactive protein concentration in a healthy Brazilian population. **Int J Cardiol**, v.97(3), p.433-438, 2004.

19. WORLD HEALTH ORGANIZATION. **Obesity: Preventing and managing the global epidemic**. Report of a WHO consultation. WHO Technical Report Series 894. cap. 2, p.6-15, Geneva, 2004.

20. SOCIEDADE BRASILEIRA DE CARDIOLOGIA. III Diretrizes Brasileiras Sobre Dislipidemias e Diretriz de Prevenção da Aterosclerose do Departamento de Aterosclerose da Sociedade Brasileira de Cardiologia. **Arq. Bras. de Cardiol,** v.77, Suplemento III, p.1-48, 2001.

21. FOOD AND NUTRITION BOARD; INSTITUTE OF MEDICINE. Selenium ___In: **Dietary reference intakes for vitamin C, vitamin E, selenium and carotenoids**. Washington, DC: National Academy Press. 2000. Cap.7, p. 284-324.

22. _____. Zinc ___In: **Dietary reference intakes for vitamin A, vitamin K, arsenic, boron, chromium, cooper, iodine, manganese, molybdenum, nickel, silicon, vanadium and zinc**. Washington, DC: National Academy Press. 2001. Cap.12, p. 1-47.

23. NATIONAL CHOLESTEROL EDUCATION PROGRAM (NCEP) III. Detection, Evaluation, and Treatment of High Blood Cholesterol in Adults (Adult Treatment Panel III). **National Institutes of Health Publication** n. 02-5215. Set, 2002.

24. SENA KCM, Arrais RF, Almeida MG, et al. Effects of zinc supplementation in patients with type 1 Diabetes. Biol Trace Elem. v 105, p.1-9, 2005

25. HAO D, XIE G, ZHANG Y, TIAN G. Determination of serum selenium by hydride generation flame atomic absorption spectrometry. Talanta, v. 43, p. 595–600, 1996.

26. FAULIN TES, SENA KCM, TELLES, AER, GROSSO DM, FAULIN EJB, ABDALLA DSP. Validation of a novel ELISA for measurement of electronegative low density lipoprotein. Clin Chem Lab Med. v 46, p.1769–75, 2008.
