# Supplementary material for: The Hypolipidemic and Pleiotropic Effects of Rosuvastatin Are Not Enhanced by Its Association with Zinc and Selenium Supplementation in Coronary Artery Disease Patients: A Double Blind Randomized Controlled Study
Source: PLoS One. 2015 Mar 18;10(3):e0119830. doi: 10.1371/journal.pone.0119830 (PMC4365008; doi:10.1371/journal.pone.0119830)
Supplement: S2 Protocol — (DOC) [file pone.0119830.s005.doc]

UNIVERSIDADE DE SÃO PAULO

FACULDADE DE CIÊNCIAS FARMACÊUTICAS

PROGRAMA DE PÓS-GRADUAÇÃO EM CIÊNCIAS DOS ALIMENTOS

STUDY PROTOCOL

**EFEITO DA SUPLEMENTAÇÃO COM MINERAIS ANTIOXIDANTES EM PACIENTES COM ATEROSCLEROSE TRATADOS COM ESTATINAS**

**PESQUISADORAS**

KARINE CAVALCANTI MAURÍCIO DE SENA

Dulcinéia Saes Parra Abdalla

São Paulo

2006

**1. INTRODUÇÃO E JUSTIFICATIVA**

A aterosclerose é caracterizada pelo acúmulo de depósitos de colesterol nos macrófagos presentes em artérias de médio e grande calibre. Este processo de deposição favorece a proliferação de certos tipos de células na parede arterial que invadem o lúmen do vaso, comprometendo o fluxo sangüíneo. As formas clínicas da aterosclerose são freqüentemente referidas como doença arterial coronariana e doença cerebrovascular, que resultam em alterações funcionais do coração e do cérebro, provocadas pela redução do fluxo sangüíneo ^(1,2)^.

Várias são as hipóteses que explicam os processos associados ao desenvolvimento da aterosclerose. A hipótese da resposta à injúria aponta a lesão vascular como o evento inicial do processo de aterosclerose. Em contraste, a teoria da resposta à retenção coloca as interações entre as lipoproteínas e a matriz como o ponto crítico da aterosclerose, enquanto que a hipótese da modificação oxidativa ressalta a importância da oxidação da LDL como o principal fator desencadeante da doença^(1)^.

Neste contexto, constata-se que a resposta inflamatória, a disfunção do endotélio vascular, bem como o estresse oxidativo, constituem importantes fatores ligados a aterogênese, intermediando a relação entre os fatores de risco e a indução da formação da placa de ateroma. A inflamação, particularmente, é o fenômeno básico que determina a instabilização da placa, por meio dos eventos inflamatórios, tais como: infiltração de leucócitos mononucleares, aumento da expressão e atividade de metaloproteinases de matriz, aumento da expressão de fatores pró-trombóticos e apoptose de células musculares lisas e endoteliais. Estes processos podem ser favorecidos pelo estresse oxidativo ^(3)^.

Por outro lado, muitas substâncias antioxidantes podem prevenir ou retardar a oxidação de substratos envolvidos nas reações de estresse oxidativo. Em relação à aterosclerose, os antioxidantes precisam proteger a vasculatura contra a ação de espécies reativas de oxigênio e nitrogênio, dentro e fora das células. Os antioxidantes enzimáticos, principalmente, a superóxido dismutase (SOD) (Cu-ZnSOD, MnSOD), catalase, glutationa peroxidase, glutationa redutase e transferases, tiol-dissulfido oxidoredutase e peroxiredoxinas estão presentes nas células da parede arterial ^(1)^.

Dentro desta perspectiva, Fields (1999)^(4)^ aponta a relação dos distúrbios de minerais com o processo aterosclerótico. As altas concentrações de ferro e cobre podem afetar diretamente o processo de oxidação da LDL. Apesar do cobre atuar como uma substância pró-oxidante em elevada concentração, por outro lado, também é constituinte da enzima Cu-ZnSOD e da ceruloplasmina, portanto o seu excesso ou deficiência pode favorecer a oxidação da LDL, hipercolesterolemia e doença coronariana^(4)^.

Em se tratando do zinco e do selênio, Henning et al (1999)^(5)^ e Alarcón-Navarro e Martinez (2000)^(6)^ afirmaram que estes minerais são importantes para a manutenção da integridade endotelial e agem como uma substância antiaterogênica, por inibir eventos relacionados ao estresse oxidativo. Outros autores mais recentemente relacionam o efeito protetor do zinco no endotélio vascular, por meio do seu papel antioxidante e de estabilizador das membranas celulares, além da sua participação na atividade do NF-kB, das enzimas caspase e do óxido nítrico sintase ^(7,10)^. O selênio, por outro lado, também pode diminuir a resposta do NF-kB ao mecanismo de pró-inflamação da célula endotelial, limitando o aparecimento da aterosclerose ^(8)^.

A deficiência do zinco e da metalotioneína, particularmente, podem aumentar os processos relacionados ao estresse oxidativo nas células endoteliais, e desde que esta alteração no zinco plasmático disponível venha afetar a condição do zinco endotelial, a deficiência deste mineral pode ser um fator de risco para formação da placa aterosclerótica ^(9)^. Já a deficiência de selênio, por sua vez, prejudica a ação da glutationa peroxidase e da tioredoxina redutase, com conseqüente acúmulo de hidroperóxidos que podem induzir a oxidação da LDL ^(7)^.

No que diz respeito ao tratamento de pacientes com aterosclerose, ressalta-se a necessidade de uma abordagem multifatorial e simultânea em relação a todos os fatores de risco, priorizando-se inicialmente a mudança do estilo de vida, incluindo a terapia nutricional, a atividade física, a abstenção do tabagismo, associadas ou não ao tratamento farmacológico específico. Para este último, recomenda-se o uso de inibidores da enzima HMG-CoA redutase por apresentarem efeitos na função vasomotora do endotélio, inflamação e trombose, além de inibirem a biossíntese do colesterol. Neste sentido, os fármacos de escolha são as estatinas (lovastatina, fluvastatina, atorvastatina, sinvastatina, pravastatina, rosuvastatina) ^(11)^.

Estas drogas, além de apresentarem a função de diminuir as concentrações de colesterol sérico, apresentam uma série de ações vasculares protetoras ou “pleiotrópicas” que incluem a melhora da função endotelial, aumento da biodisponibilidade do ^•^NO, estabilização da placa aterosclerótica, inibição da resposta inflamatória e ações imunomoduladoras, reduzindo a incidência de infarto em pacientes tratados com inibidor da HMG-CoA redutase ^(12)^.

Considerando a importância dos metais (ex.: ferro, cobre, zinco, selênio) nos processos oxidativos, alguns estudos mostram a relação entre estes elementos e o uso das estatinas. Leonhardt et al (1997)^(13)^ concluíram que o tratamento de pacientes hipercolesterolêmicos com fluvastatina durante 8 semanas, resultou em redução de colesterol total, de triglicerídeos, e de zinco plasmático, ressaltando a importância deste último, tendo em vista que o zinco está diretamente relacionado com a atividade da enzima CuZnSOD dismutase.

Corroborando com estes resultados, Ghayour-Mobarhan et al (2005)^(14)^, avaliaram durante 4 meses, o efeito da terapia com simvastatina e atorvastatina em pacientes dislipidêmicos (estudo caso-controle) e observaram que após o tratamento, houve uma diminuição significativa do colesterol total, LDL-colesterol, zinco sérico, cobre, ceruloplasmina e proteína C reativa; sem alteração do selênio sérico, da razão cobre/ceruloplasmina e da glutationa peroxidase no grupo estatina. O estudo demonstra que os níveis desses minerais traços podem ser afetados significantemente pelo tratamento com doses de estatinas rotineiramente utilizadas para tratamento clínico.

Moosmann e Behl (2004)^(15)^ ressaltaram que alguns dos efeitos colaterais do uso das estatinas, como miopatia, rabdomiólise e polineuropatia, assemelham-se aos da deficiência de selênio. Os autores sugerem que o efeito negativo das estatinas na síntese de selenoproteínas parece explicar algumas das colateralidades das estatinas, especialmente a miopatia.

Diante destas considerações, o presente estudo se propõe avaliar o efeito da suplementação com minerais antioxidantes, zinco e selênio, em pacientes com doença arterial coronariana sob tratamento com estatinas, identificando o efeito potencial destes minerais concomitante ao uso das estatinas sobre os marcadores do estresse oxidativo, de inflamação e enzimas antioxidantes.

**2. OBJETIVOS**

**2.1 Objetivo geral:**

- Avaliar o efeito da suplementação oral de zinco e selênio sobre o estresse oxidativo em pacientes com doença arterial coronariana (DAC) apresentando angina estável e tratados com estatinas.

**2.2 Objetivos específicos:**

- Realizar a avaliação do estado nutricional antropométrico e a dieta consumida pelos pacientes;

- Identificar o estado nutricional dos pacientes em relação ao zinco e selênio antes e após a suplementação;

- Avaliar o perfil lipídico, LDL oxidada, marcadores de inflamação e enzimas antioxidantes nos grupos estudados antes e após a suplementação;

- Correlacionar as concentrações de zinco e selênio com o perfil lipídico, LDL-, marcadores de inflamação e enzimas antioxidantes nos grupos estudados.

**3. PLANO DE TRABALHO - CRONOGRAMA DE EXECUÇÃO**

| Aug | Sep | Oct | Nov | Dec |  |
| --- | --- | --- | --- | --- | --- |
|  |  |  |  |  | **Submissão do Projeto do Comitê de Ética** |
|  |  |  |  |  | **Aquisição de Material** |
|  |  |  |  |  | **Padronização de Metodologias**  * Avaliação e coletas de dados para:  Análises antropométricas e dietéticas  Análise do perfil lipídico  Análise bioquímica do zinco e selênio  Análise dos marcadores do estresse oxidativo e inflamação  Análise das enzimas antioxidantes |
|  |  |  |  |  |  |

| Jan | Fev | Mar | Apr | May | Jun | Jul | Aug | Sep | Oct | Nov | Dec |  |
| --- | --- | --- | --- | --- | --- | --- | --- | --- | --- | --- | --- | --- |
|  |  |  |  |  |  | **Padronização de Metodologias** | | | | | |  |
|  |  |  |  |  |  | **Preparo /aquisição de suplementação e medicação** | | | | | |  |
|  |  |  |  |  |  |  | **Contatos com o ambulatório para início das coletas** | | | | |  |
|  |  |  |  |  |  |  |  |  |  |  |  | **Seleção de participantes**  **1ª Avaliação (Coleta de dados)** |
|  |  |  |  |  |  |  |  |  |  |  |  |  |
|  |  |  |  |  |  |  |  |  |  |  |  | **Início da suplementação** |
|  |  |  |  |  |  |  |  |  |  |  |  |  |

| Jan | Fev | Mar | Apr | May | Jun | Jul | Aug | Sep | Oct | Nov | Dec |  |
| --- | --- | --- | --- | --- | --- | --- | --- | --- | --- | --- | --- | --- |
|  |  |  |  |  |  | **2ª Avaliação (coleta de dados) pós-suplementação** | | | | | |  |
|  |  |  |  |  |  |  | | | | | |  |
|  |  |  |  |  |  |  |  |  |  |  |  | **Análises Bioquímicas**  **Análises dos recordatórios 24h.** |
|  |  |  |  |  |  |  |  |  |  |  |  |  |

| Jan | Fev | Mar | Apr | May | Jun | Jul | Aug | Sep | Oct | Nov | Dec |  |
| --- | --- | --- | --- | --- | --- | --- | --- | --- | --- | --- | --- | --- |
|  |  |  | **Descegamento do estudo – análises estatísticas**  **Tabulação dos dados**  **Qualificação** | | | | | | | | |  |
|  |  |  |  |  |  |  |  | **Elaboração do trabalho final, defesa da tese e redação de artigos** | | | |  |

**4. MATERIAL E MÉTODOS**

**4.1. Protocolo Experimental**

O estudo caracteriza-se por um ensaio clínico randomizado duplo-cego que será desenvolvido com um grupo de pacientes adultos e idosos com o diagnóstico clínico de doença arterial coronariana submetidos à angioplastia, atendidos no Serviço de Hemodinâmica do Hospital Universitário Onofre Lopes (HUOL) da UFRN, Natal/ RN, durante o período de agosto de 2007 à junho de 2008 (Fluxograma 1).

Os pacientes serão recrutados no referido Serviço Ambulatorial, triados pela pesquisadora e médica responsável, considerando os seguintes critérios de inclusão: pacientes adultos e idosos de ambos os gêneros com diagnóstico de aterosclerose coronariana por angiografia com estenose > 70% em uma ou mais artérias. Serão considerados como critérios de exclusão a presença de complicações cardíacas graves ou outras doenças como as hematológicas, auto-imunes, hepatopatias, insuficiência renal, neoplasias, diabetes mellitus, infecções associadas, pós-operatório, uso de antiácidos, antibióticos e suplementos vitamínicos-minerais, etilismo e tabagismo.

O tamanho amostral ^(16)^ foi calculado assumindo-se 90% de poder estatístico para a detecção de uma diferença de, no mínimo, 0,075nmol/mg entre as médias da LDL oxidada do grupo experimental e grupo controle. Assumiu-se que a diferença de 0,075nmol/mg como sendo clinicamente significativa baseada em estudos prévios^(17)^. Os parâmetros como média e variância populacional foram baseados em estudos com amostras robustas^(17,18)^ que avaliaram populações brasileiras semelhantes aos grupos a serem estudados. As simulações revelaram um tamanho amostral de 76 indivíduos (38 experimentais e 38 controles) para a detecção de um poder estatístico de 90% e um nível de significância de 0.05.

Os pacientes com aterosclerose que formarão os grupos serão escolhidos por meio de um sorteio aleatório. O grupo experimental será formado por pacientes com aterosclerose que serão submetidos ao tratamento com estatina + suplemento zinco e selênio e o grupo controle composto por pacientes que receberão estatina + placebo.

Os pacientes do grupo aterosclerose serão tratados com rosuvastatina do Laboratório Astra|Zeneca® na dosagem de 10 mg/dia. Já a suplementação de zinco e selênio será preparada a partir da matéria prima Zinco Aminoácido Taste Free® e Selênio Complexo® da Albion Laboratories Incorporation e a fórmula farmacêutica manipulada de acordo com as recomendações do fabricante.

Para a determinação das doses dos mineras serão consideradas as recomendações das DRI’s (2001) que definem a ingestão diária máxima de zinco e selênio para adultos de ambos os sexos acima de 50 anos de 40mg/dia e 400µg/dia, respectivamente. Portanto, considerando os resultados de estudos prévios de avaliação da ingestão de zinco e selênio em pacientes portadores de doenças crônicas não transmissíveis atendidos em Ambulatório da Cidade de Natal, que revelaram uma ingestão média de 7mg/dia de zinco e 50µg/dia de selênio, as doses utilizadas serão 30mg/Zn/dia e 300µgSe/dia. Os pacientes serão acompanhados mensalmente no Ambulatório para monitoração do uso da droga+suplemento, esclarecimento de dúvidas e identificação de intercorrências ou reações adversas.

A coleta de dados será realizada no primeiro momento e após 4 meses do tratamento e constará dos seguintes parâmetros: avaliação do estado nutricional relativo ao zinco e selênio, selenoproteínas P, perfil lipídico, LDL minimamente oxidada (LDL eletronegativa), marcadores da inflamação e enzimas antioxidantes. A avaliação antropométrica e dietética será realizada para a caracterização da população quanto ao estado nutricional antropométrico e dietético.

**4.1.1 Aspectos Éticos**

O estudo será realizado em conformidade com as diretrizes regulamentadoras de pesquisas envolvendo seres humanos, que constam na Resolução 196/96 do Conselho Nacional de Saúde (Ministério da Saúde, 1996) e será analisado pelo Comitê de Ética em Pesquisa do Hospital Universitário Onofre Lopes da UFRN e da Faculdade de Ciências Farmacêuticas da USP. Os dados somente serão coletados após a permissão do paciente, por meio da assinatura do termo de consentimento livre e esclarecido aplicado pelo pesquisador responsável, mediante informações sobre os objetivos, riscos e benefícios do estudo. A pesquisa será encerrada após a conclusão das etapas definidas no cronograma de atividades.

O voluntário será informado sobre a formação de grupos experimental e controle (placebo), e o mesmo poderá retirar seu consentimento a qualquer momento e deixar de participar da pesquisa, sem que isso traga qualquer prejuízo no seu acompanhamento. A participação no estudo não acarretará custos para o paciente e não será disponível nenhuma compensação financeira adicional, porém em caso de haver gastos com transporte, alimentação, poderá ser oferecida a quantia referente a estes gastos. Os dados obtidos a partir deste trabalho somente serão utilizados para fins científicos e será assegurada à proteção da imagem e identidade do participante.

Os riscos possíveis associados à participação nesta pesquisa são aqueles referentes à coleta de sangue, por exemplo, sangramentos, hematomas leves. Para tanto, as coletas serão realizadas no Laboratório do Hospital Universitário Onofre Lopes por profissionais treinados e qualquer intercorrência será encaminhada para o Serviço de Pronto-Atendimento do Hospital. A medicação (rosuvastatina), prescrita pelo médico, segundo informações do fabricante pode causar as reações: - **Raras:** efeitos musculares desagradáveis e reações alérgicas graves, que podem afetar as vias respiratórias; - **Incomuns:** reações leves que desaparecem rapidamente como coceira, vermelhidão e reações alérgicas na pele; - **Comuns:** dor de cabeça, dores musculares, dor de estômago, sensação geral de fraqueza, prisão de ventre, tontura e mal-estar. O uso dos suplementos de zinco e selênio nas dosagens recomendadas não apresenta efeitos colaterais. Os pacientes serão orientados a comunicar o médico e/ou pesquisadora responsável quaisquer intercorrências para que sejam tomadas as devidas providências.

Os resultados deste estudo oferecerão esclarecimentos sobre o estado nutricional antropométricos e dietéticos do pacientes com aterosclerose, bem como as alterações metabólicas do zinco e selênio em pacientes sob uso da rosuvastatina, considerando o importante papel destes minerais na defesa antioxidante e nos mecanismos inflamatórios, de forma que sejam direcionadas condutas médico-nutricionais mais específicas para o tratamento deste grupo de pacientes.

**FLUXOGRAMA 1**

**ENSAIO CLÍNICO RANDOMIZADO DUPLO-CEGO**

**Casuística**

Pacientes com aterosclerose

Serviço de Hemodinâmica do HUOL/UFRN

- Aplicação do Termo de consentimento

- Avaliação antropométrica

- Entrega do Formulário para Registro de 3 dias

- Agendamento para coletas de sangue e retorno do Registro Alimentar.

**Sorteio Randômico**

**Grupo 1**

Estatina + Supl Zn e Se

**Grupo 2**

Estatina + Placebo

Perfil

Lipídico/

ALT/ AST

PCR

Ultra-sensível

Zinco

Plasma/

eritrócito

Selênio Plasma/

eritrócito

Selenoproteinas - P

CuZnSOD

GPx

LDL-

E-selectina

**Avaliações**

**T1 e T2**

**Segmento 4 meses**

IL-8

Lab. Análises Clínicas – HUOL-UFRN

UFRN

Lab Multi UFRN

Lab Nut. Minerais B14 - USP

Laboratório de Bioquímica Clínica – B 17 - USP

**4.2. Avaliação Antropométrica**

Altura e peso serão medidos usando uma estadiômetro portátil com plataforma (WSC® Cardiomed, Curitiba, Brazil) e uma balança solar digital MEA-03140 (Tanita, Arlington Heights, IL, USA), respectivamente. Estes valores serão utilizados para calcular o índice de massa corporal (IMC) de acordo com WHO (2004)^(19)^. Será realizada também a medida da circunferência abdominal (CA) de acordo com WHO (2004)^(19)^.

**4.3. Avaliação Dietética**

Será utilizado o método do Registro Alimentar de 3 dias, que consistirá na anotação de todos os alimentos consumidos pelos pacientes e controles durante 3 dias consecutivos (2 dias durante a semana e 1 dia no final de semana), aplicado no primeiro momento da pesquisa. Os dados coletados serão analisados com o auxílio de programas computadorizados específicos para análises de dietas e a avaliação da adequação de energia, macronutrientes, fibra, zinco e selênio será feita considerando as recomendações da Sociedade Brasileira de Cardiologia (2001)^(20)^ e DRI’s 2000^(21)^ e 2001^(22)^.

**4.4. Coleta do Material Biológico**

As amostras de 30mL de sangue de ambos os grupos serão coletadas com material descartável, pela manhã, após um período de 12 a 14 horas de jejum. Em seguida, as amostras serão distribuídas em tubos, com ou sem anticoagulantes, submetidas a procedimentos específicos para cada análise e armazenadas à temperatura de -80^o^C para posterior análises, conforme quadro abaixo:

| **TUBO** | **AMOSTRA** | **ANTICOAGULANTE** | **ANÁLISES** |
| --- | --- | --- | --- |
| 1 | 6 mL | Sem  Anticoagulante | Perfil Lipídico, AST, ALT  PCR Ultra-sensível |
| 2 | 6 mL | Citrato de sódio a 30% | Zinco plasmático, Zinco Eritrocitário  Hemoglobina Eritrocitária |
| 3 | 10mL | EDTA | Selênio plasmático , Selênio Eritrocitário, LDL -, selenoproteína-P |
| 4 | 8 mL | Heparina | CuZnSOD, GPx,  E-Selectina e IL-8 |

**4.5. Análises Bioquímicas**

As análises serão realizadas no Laboratório de Análises Clínicas do HUOL/UFRN, Laboratório Multidisciplinar de Pesquisa do Departamento de Farmácia da UFRN, Laboratório de Nutrição e Minerais e Bioquímica Clínica da Faculdade de Ciências Farmacêuticas da Universidade de São Paulo.

**4.5.1. Avaliação do Perfil Lipídico e Função Hepática**

O perfil lipídico será avaliado a partir da determinação das concentrações plasmáticas de colesterol total (CT), triglicerídios (TG), LDL e HDL. Para a medida das concentrações plasmáticas de CT, TG e HDL será utilizado o método enzimático colorimétrico, por meio dos kits reagentes da Labtest®. A determinação da fração LDL será realizada por meio da Fórmula de Friedewald, LDL = CT – HDL + (TG/5), quando o TG for < 400mg/dL. Será utilizada também a medida do colesterol não-HDL (CT – HDL) que representa a soma das frações LDL e VLDL ^(23)^.

Para avaliação da função hepática será feita a determinação das concentrações séricas de AST, ALT e GGT, utilizando-se os kits reagentes Labtest®. Pelo método cinético UV-IFCC. Os intervalo de referência considerados normais serão de 14 a 50U/L e 11 a 39U/L, respectivamente.

**4.5.2. Avaliação bioquímica do zinco e selênio**

Toda vidraria e recipientes plásticos utilizados durante a coleta de sangue e análises de minerais serão cuidadosamente desmineralizados em banho de ácido nítrico a 30 % durante pelo menos 12 h e enxaguados 10 vezes utilizando-se água ultra-pura (Milli - Q para minimizar a contaminação de minerais. O soro e o plasma será separado por centrifugação durante 15 min a 3500 rpm a 4^o^C. Os eritrócitos obtido serão lavados três vezes com 5mL de solução salina 0,9% , lentamente homogeneizada por inversão e centrifugado novamente a 10.000rpm durante 10 minutos (centrífuga SIGMA ® 2K15-Alemanha), 4^o^C, e o sobrenadante será descartado. Após a centrifugação final, a solução salina será aspirada e a massa de eritrócitos será cuidadosamente extraída utilizando uma micropipeta, transferido para tubos de eppendorf desmineralizados e armazenados para as análises posteriores de zinco e de hemoglobina . No caso de ensaios que não serão realizados no dia da coleta de sangue, as alíquotas serão armazenadas a -80^o^C.

Concentrações de zinco no plasma e eritrócitos serão quantificados por meio de espectrofotometria de absorção atômica Spectra Varian AA- 240 ( Varian Medical Systems, Inc., Milpitas , CA , EUA). As concentrações de zinco no plasma serão determinadas de acordo com os métodos que foram anteriormente descritos ^(24)^ . As análises de selênio no plasma e eritrócitos serão realizadas por meio de espectrometria de absorção atômica por meio do aparelho Hitachi Z - 5000 (Tóquio, Japão) com a geração de hidreto acoplada a uma célula de quartzo, utilizando técnicas anteriormente descritas ^(25)^ . Seronorm ™ Oligoelementos Soro L - 1 ( Sero AS , Billingstad , Noruega ) vai ser utilizado como uma referência durante a análise de zinco e selênio .

**4.5.3. Determinação da Selenoproteína-P**

A determinação de selenoproteína – P será realizada por intermérdio de ensaio de ELISA, utilizando anticorpo monoclonal Anti-Selenoprotein-P (LF-MA0141) do Labarotório LabFrontier®.

**4.5.4. Avaliação da LDL oxidada (LDL^-^)**

Ensaios de elisa serão utilizados para avaliar as concentrações de LDL(-) de acordo com os métodos descritos por Faulin et al.^(26)^.

**4.5.5. Avaliação de Glutationa Peroxidase e CuZnSOD dismutase**

A atividade da enzima GPx sera avaliada usando o kit RANSEL® kit (RS504; Randox Laboratory, San Francisco, CA, USA). O material de referência Ransel Control (SC692; Randox Laboratory) será utilizado como controle. A atividade da superóxido dismutase sera medida por meio do kit RANSOD® (SD125; Randox Laboratory). Material de referência Ransod Control (SD126; Randox Laboratory) sera utilizado como referência.

**4.5.6. Avaliação dos marcadores da inflamação**

**4.5.6.1. Determinação da Proteína C Reativa (PCR)**

As concentrações de PCR serão quantificadas no soro em níveis de ultra-sensibilidade utilizando-se o kit da Biotécnica (CAT BT-20.017.00)® , seguindo as orientações propostas no kit e os dados apresentados em mg/dL.

**4.5.6.2. Determinação da E-selectina**

A dosagem plasmática da E-selectina será realizada por meio do kit Human sE-selectin Immunoassay da R&D Systems(BBE 2B)®, de acordo com o protocolo definido pelo fabricante. As leituras serão realizadas em 450nm no luminômetro para microplacas e os valores apresentados em ng/mL.

**4.5.6.3. Determinação da IL-8**

A determinação da IL-8 será feita utilizando-se o kit Human CXCL8/IL-8 da R&D Systems(D8000C)®, considerando as orientações do kit e as leituras realizadas em 450nm no luminômetro para microplacas. Os dados serão expressos em pg/mL.

**5. FORMA DE ANÁLISE DOS RESULTADOS**

Os dados serão apresentados como média ± desvio padrão ou mediana (intervalo interquartil) quando apropriado. Para a comparação inter-grupo (experimental vs controle) as variáveis com distribuição normal ou próxima da normal serão comparadas com o teste “t” de Student para amostras independentes. Entretanto, quando as variáveis apresentarem distribuição assimétrica, o teste não-paramétrico de Mann-Whitney será aplicado. Será utilizado o teste exato de Fisher para variáveis categóricas ou binárias, e suas extensões por 2 × k tabelas de contingência, para compararação dos dados. Para comparações intergrupos, as diferenças (δ) serão calculadas considerando: δ = grupo Δsupplemented - grupo Δplacebo. Para essas análises, o teste t de Student para amostras independentes será utilizado para as variáveis com distribuição normal, enquanto o U-Mann-Whitney será aplicado para as variáveis ​​com distribuição assimétrica. As estimativas de δ, serão ajustadas para potenciais variáveis ​​de confusão, e será calculado por vários modelos de regressão linear.

FINANCIAMENTO:

Este projeto será submetido aos editais de agências de fomento, tais como CNPq ou FAPESP – Fundo de Amparo à Pesquisa do Estado de São Paulo para financiamento. Serão também consideradas verbas dos Laboratórios onde serão realizadas as análises.

REFERÊNCIAS BIBLIOGRÁFICAS

1. STOCKER, R., KEANEY, J.F. Role of oxidative modifications in atherosclerosis. **Physiol Rev**, v. 84, p.1381-1478, 2004.

2. SCHOEN, F.J. **Os vasos sangüíneos**. In: KUMAR.V, ABBAS A.K., FAUSTO, N. Robbins e Cotran: Patologia – bases patológicas das doenças. Rio de Janeiro:Elsevier. 2005, cap.11, 537-581p.

3. SOUZA, H.P.; LAURINDO, F.R.M. Estresse oxidativo e ruptura da placa aterosclerótica. **Rev Soc Cardiol Estado de São Paulo**, v.12(4), p.584-594, 2002.

4. FIELDS, M. Role os trace elements in coronary heart disease. **British J Nutr**, v. 81, p.85-86, 1999.

5. HENNING, B., MEERARANI, P.; TOBOREK, M.; McCLAIN, C.J. Antioxidant-like properties of zinc in activated endothelial cells. **J Am Coll Nutr**, v.18(2), p.152-158, 1999.

6. ALARCÓN-NAVARRO, M., MARTINEZ, M.C.L. Essenciality of selenium in the human body: relationship with diferent disease. **Sci Total Environ,** v. 249, p.347-371, 2000.

7. BEATTIE, J.H.; KWUN, I.S. Is zinc deficiency a risk factor for atherosclerosis. **British J Nutr,** v. 91, p.177-181, 2004.

8. [Zhang F](http://www.ncbi.nlm.nih.gov/entrez/query.fcgi?db=pubmed&cmd=Search&itool=pubmed_Abstract&term="Zhang+F"%5BAuthor%5D), [Yu W](http://www.ncbi.nlm.nih.gov/entrez/query.fcgi?db=pubmed&cmd=Search&itool=pubmed_Abstract&term="Yu+W"%5BAuthor%5D), [Hargrove JL](http://www.ncbi.nlm.nih.gov/entrez/query.fcgi?db=pubmed&cmd=Search&itool=pubmed_Abstract&term="Hargrove+JL"%5BAuthor%5D), [Greenspan P](http://www.ncbi.nlm.nih.gov/entrez/query.fcgi?db=pubmed&cmd=Search&itool=pubmed_Abstract&term="Greenspan+P"%5BAuthor%5D), [Dean RG](http://www.ncbi.nlm.nih.gov/entrez/query.fcgi?db=pubmed&cmd=Search&itool=pubmed_Abstract&term="Dean+RG"%5BAuthor%5D), [Taylor EW](http://www.ncbi.nlm.nih.gov/entrez/query.fcgi?db=pubmed&cmd=Search&itool=pubmed_Abstract&term="Taylor+EW"%5BAuthor%5D), [Hartle DK](http://www.ncbi.nlm.nih.gov/entrez/query.fcgi?db=pubmed&cmd=Search&itool=pubmed_Abstract&term="Hartle+DK"%5BAuthor%5D). Inhibition of TNF-alpha induced ICAM-1, VCAM-1 and E-selectin expression by selenium. **Atherosclerosis**, v.161(2), p. 381-386, 2002.

9. HENNING, B., MEERARANI, P.; RAMADASS, P.; TOBOREK, M.; MALECKI, A.; SLIM, R.; McCLAIN, C.J. Zinc nutrition and apoptosis of vascular endothelial cells: implications in atherosclerosis. **Nutrition**, v.15, p. 744-748, 1999.

10. ALISSA, E.M; BAHJRI, S.M.; AHMED, W.H.; AL-AMA, N.; FERNS, G.A.A. Trace elements status in Saudi patients with established atherosclerosis. **J Trace Elem Med Biol**, in press, 2006.

11. KINLAY, S. Potential vascular benefits of statins. **Am J Med**, v. 118(12A), p.625-675, 2005.

12. ENDRES, M. Statins: Potential and indications in inflammatory conditions. **Atherosclerosis** Supplements., v.7, p.31-35, 2006.

13. LEONHARDT, W.; KURKTSCHIEV, W.L.; MEISSNER, D.; LATTKE, P; ABLETSHAUSER, C.; WEIDINGER, G.; JAROSS, W.; HANEFELD, M. Effects os fluvastatin therapy on lipids, antioxidants, oxidation of low density lipoproteins and trace metals. **Eur J Clin Pharmacol**, v. 53, p.65-69, 1997.

14. GHAYOUR-MORBARHAN,M.; LAMB, D.J.; TAYLOR, A.; VAIDYA, N.; LIVINGSTONE, C.; WANG, T; FERNS, G.A.A. Effect of statin therapy on serum trace element status in dyslipidemic subjetcts. **J Trace Elem Med Biol**, v.19, p.61-67, 2005.

15. MOOSMAN, B.; BEHL,C. Selenoprotein synthesis and side-effects of statins. **Lancet**, v. 363, p.892-894, 2004.

16. GOTTLEIB, M.G; SCHWANKE, C.H.; SANTOS, A.F; JOBIM, P.F.; MUSSEL, D.P.; CRUZ,I.B. Association among oxidized LDL levels, MnSOD, apolipoprotein E polymorphisms, and cardiovascular risk factors in a south Brazilian region population. **Genet Mol Res**, v.4(4), p. 691-703, 2005.

17. LUZ, P.L.; CESERA, F.H; FAVARATO, D. CERQUEIRA, E.S.Comparasion of serum lipids values in patients with coronary artery disease at <50, 50 to 59, 60 to 69, and >70 years of age. **Am J Cardiol**, v. 96(12), p. 1640-1643, 2005.

18. ARAÚJO, F., PEREIRA, A.C., LATORRE, M.R., KRIEGER, J.E., MANSUR, A.J. High-sensitivity C reative protein concentration in a healthy Brazilian population. **Int J Cardiol**, v.97(3), p.433-438, 2004.

19. WORLD HEALTH ORGANIZATION. **Obesity: Preventing and managing the global epidemic**. Report of a WHO consulation. WHO Technical Report Series 894. cap. 2, p.6-15, Geneva, 2004.

20. SOCIEDADE BRASILEIRA DE CARDIOLOGIA. III Diretrizes Brasileiras Sobre Dislipidemias e Diretriz de Prevenção da Aterosclerose do Departamento de Aterosclerose da Sociedade Brasileira de Cardiologia. **Arq. Bras. de Cardiol,** v.77, Suplemento III, p.1-48, 2001.

21. FOOD AND NUTRITION BOARD; INSTITUTE OF MEDICINE. Selenium ___In: **Dietary reference intakes for vitamin C, vitamin E, selenium and carotenoids**. Washington, DC: National Academy Press. 2000. Cap.7, p. 284-324.

22. _____. Zinc ___In: **Dietary reference intakes for vitamin A, vitamin K, arsenic, boron, chromium, cooper, iodine, manganese, molybdenum, nickel, silicon, vanadium and zinc**. Washington, DC: National Academy Press. 2001. Cap.12, p. 1-47.

23. NATIONAL CHOLESTEROL EDUCATION PROGRAM (NCEP) III. Detection, Evaluation, and Treatment of High Blood Cholesterol in Adults (Adult Treatment Panel III). **National Institutes of Health Publication** n. 02-5215. Set, 2002.

24. SENA KCM, Arrais RF, Almeida MG, et al. Effects of zinc supplementation in patients with type 1 Diabetes. Biol Trace Elem. v 105, p.1-9, 2005

25. HAO D, XIE G, ZHANG Y, TIAN G. Determination of serum selenium by hydride generation flame atomic absorption spectrometry. Talanta, v. 43, p. 595–600, 1996.

26. FAULIN TES, SENA KCM, TELLES, AER, GROSSO DM, FAULIN EJB, ABDALLA DSP. Validation of a novel ELISA for measurement of electronegative low density lipoprotein. Clin Chem Lab Med. v 46, p.1769–75, 2008.
